# Achados Ecocardiográficos Anormais em Pacientes Internados com Covid-19: Uma Revisão Sistemática e Metanálise

**DOI:** 10.36660/abc.20210485

**Published:** 2022-07-07

**Authors:** Silvio Henrique Barberato, Eduardo G. Bruneto, Gabriel S. Reis, Paula Rauen Franco de Oliveira, Alexandre F. Possamai, Odilson Silvestre, Miguel M. Fernandes Silva

**Affiliations:** 1 Centro de Diagnóstico Cardiovascular Curitiba PR Brasil CardioEco Centro de Diagnóstico Cardiovascular, Curitiba, PR – Brasil; 2 Quanta Diagnóstico, Ecocardiografia Curitiba PR Brasil Quanta Diagnóstico, Ecocardiografia,Curitiba, PR – Brasil; 3 Universidade Federal do Acre Rio Branco AC Brasil Universidade Federal do Acre, Rio Branco, AC – Brasil; 4 Universidade Federal do Paraná Curitiba PR Brasil Universidade Federal do Paraná, Curitiba, PR – Brasil; 5 Pontifícia Universidade Católica do Paraná Curitiba PR Brasil Pontifícia Universidade Católica do Paraná, Curitiba, PR – Brasil

**Keywords:** Ecocardiografia, Covid-19, Miocardite, Função Ventricular Esquerda

## Abstract

**Fundamentos:**

A doença do coronavírus 2019 (Covid-19) pode levar à insuficiência respiratória grave e lesão cardíaca aguda, mas não está claro com que frequência ela pode causar disfunção cardíaca.

**Objetivos:**

Nesta revisão sistemática, nosso objetivo foi resumir os principais achados ecocardiográficos em pacientes com Covid-19.

**Métodos:**

Conduzimos uma busca sistemática nos bancos de dados PUBMED, EMBASE, LILACS e Cochrane, além de artigos não pulicados ( *preprints* ) no MedRxiv e Scielo desde o início até 21 de julho de 2021. Foram incluídos estudos que apresentaram dados ecocardiográficos de pacientes com Covid-19. Características demográficas, doença cardiovascular (DCV) prévia, e achados ecocardiográficos foram extraídos dos estudos. Realizamos uma metanálise de proporções para estimar os principais achados ecocardiográficos. O nível de significância foi p<0,05.

**Resultados:**

Do total de 11 233 estudos, 38 preencheram os critérios de inclusão e foram incluídos na metanálise. A proporção estimada de disfunção sistólica do ventrículo esquerdo (VE) foi 25% (IC95%: 19, 31; I^2^ 93%), *strain* longitudinal global anormal 34% (IC95% 23, 45; I^2^ 90%), disfunção sistólica do ventrículo direito (VD) 17% (IC95% 13, 21; I^2^ 90%), derrame pericárdico 17% (IC95%: 9, 26; I^2^ 97%), e hipertensão pulmonar 23% (IC95%: 15, 33, I^2^ 96%). Disfunção sistólica do VE foi diretamente associada com prevalência de ecocardiograma anormal prévio nos estudos (p<0,001). A proporção de pacientes em ventilação mecânica, indicando gravidade da doença, não explicou a heterogeneidade nas proporções de disfunção do VE (p=0,37).

**Conclusão:**

Entre os pacientes internados com Covid-19, a disfunção ventricular esquerda foi descrita em um quarto dos pacientes, com menores proporções de disfunção do ventrículo direito, derrame pericárdico e hipertensão pulmonar. No entanto, houve uma proporção mais alta de disfunção do VE nos estudos que relataram presença de doença cardíaca prévia, sugerindo que a disfunção cardíaca era predominantemente pré-existente.

## Introdução

A Doença do Coronavírus 2019 (Covid-19), causada pelo novo coronavírus da síndrome respiratória aguda grave (SARS-CoV-2), pode resultar em insuficiência respiratória grave e lesão cardíaca aguda. Níveis elevados de troponina e exames de imagem mostrando uma função cardíaca anormal foram associados a um pior prognóstico em pacientes com Covid-19 aguda.^1^ O pior prognóstico pode resultar de uma combinação de fatores relacionados à doença, tais como virulência e resposta inflamatória, e fatores relacionados ao paciente, como fatores de risco cardiovascular e doença cardiovascular (DCV) pré-existentes. Por isso, tem-se recomendado avaliar a função cardíaca por ecocardiografia transtorácica (ETT) para o direcionamento no manejo dos pacientes com sintomas cardiovasculares novos ou progressivos, instabilidade hemodinâmica, e níveis elevados de biomarcadores.^2^

No início da pandemia, existiram relatos infundados da ocorrência de insuficiência cardíaca (IC) e miocardite fulminante em pacientes com Covid-19.^3 , 4^ Estudos utilizando ressonância magnética cardíaca mostraram que evidência de inflamação do miocárdio em atletas de elite, que recentemente se recuperaram de Covid-19 era comum, porém sem significância clínica.^5^ Contudo, estudos mais recentes mostraram que a ocorrência de miocardite é bem menos comum (menos que 2%) que a sugerido anteriormente, mesmo em pacientes com níveis elevados de troponina.^6 , 7^ Também não se sabe com que frequência a Covid-19 afeta a função cardíaca, seja por lesão direta no miocárdio ou por aumento da resistência pulmonar quando os pulmões estão gravemente comprometidos. Estudos ecocardiográficos têm apresentado achados variáveis de disfunção ventricular direita e esquerda; por exemplo, a disfunção sistólica do ventrículo esquerdo (VE) tem sido identificada em menos de 10% dos pacientes em alguns estudos, e em quase 40% em outros.^8 , 9^ Ainda faltam estudos amplos, mostrando estimativas precisas da incidência de disfunção cardíaca significativa, sua importância clínica, e fatores de risco associados. Dado o risco de contaminação dos profissionais de saúde, o uso de ecocardiografia deve ser criticamente baseado nos benefícios para o paciente.^10^ Portanto, nosso objetivo foi resumir os principais achados ecocardiográficos de pacientes com Covid-19 por meio de uma revisão sistemática e metanálise.

## Métodos

### Delineamento do estudo e critérios de elegibilidade

Nós conduzimos uma revisão sistemática, seleção de estudos e metanálise de proporções de acordo com as recomendações PRISMA para metanálise.^11^ Incluímos todos os estudos com pelo menos 10 participantes, descrevendo os achados ecocardiográficos em pacientes hospitalizados por Covid-19, publicados em inglês, português ou espanhol, até 21 de julho de 2021. Estudos que não apresentaram achados ecocardiográficos foram excluídos. Também excluímos resumos não publicados, estudos faltando informações clínicas basais dos pacientes, ou dados ecocardiográficos insuficientes para se obter o número de participantes com função ou estrutura cardíaca anormal.

### Fontes e busca de informações

Realizamos uma busca sistemática nos bancos de dados PUBMED, EMBASE, LILACS e Cochrane (CENTRAL). Também buscamos artigos no repositório de artigos não publicados ( *preprints* ) nos bancos de dados do MedRxiv (https://www.medrxiv.org/) e Scielo. Para a busca, usamos os termos “Covid-19”, “SARS CoV 2”, “Coronavirus infection”, “Heart Diseases” e “Echocardiography” como descritores (Medical Subject Headings – MeSH) ou conceito suplementar ( *supplementary concept* ) e sinônimos como texto livre nos títulos e resumos para aumentar a sensibilidade. A estratégia de busca de artigos completos está descrita no material suplementar (Tabela Suplementar 3).

### Seleção de estudos e extração de dados

Combinamos os resultados de busca de cada banco de dados usando o programa EndNote, removendo os estudos duplicados. Quatro autores (EB, GR, PO, AP) avaliaram independentemente títulos e resumos para excluir artigos sem relevância. Os textos completos de artigos potencialmente relevantes foram examinados e os estudos que preencheram os critérios de elegibilidade foram selecionados. Artigos diferentes, derivados dos mesmos estudos foram associados, e aquele com a maior amostra foi selecionado. Discrepâncias foram solucionadas por consenso. As referências dos artigos de revisão foram examinadas quanto a outros estudos, e aqueles considerados elegíveis foram incorporados na metanálise.

Os seguintes dados foram extraídos dos estudos: nomes dos autores, mês de publicação, ecocardiograma anormal dos pacientes, tamanho amostral dos pacientes submetidos à ecocardiografia, e número de indivíduos com disfunção sistólica no VE, disfunção sistólica no ventrículo direito (VD), hipertensão pulmonar, e derrame pericárdico. As definições de cada estudo para cada anormalidade ecocardiográfica encontram-se detalhadas na Tabela Suplementar 2. Quando a disfunção sistólica não foi claramente definida pelos autores, nós adotamos uma fração de ejeção do VE (FEVE) inferior a 50%. *Strain* longitudinal global (GLS) anormal foi definido como inferior a 18%. Disfunção ventricular direita foi determinada conforme definição usada em cada estudo e, em sua ausência, adotamos a excursão sistólica do plano anular tricúspide (TAPSE) inferior a 17 mm e/ou Doppler tecidual da parede livre do VD (S’) menor que 9,5 cm/s. Hipertensão pulmonar foi definida por velocidade de regurgitação tricúspide acima de 2,8 cm/s, tempo de aceleração pulmonar abaixo de 100 ms e/ou pressão sistólica na artéria pulmonar (PSAP) acima de 35mmHg. (Tabela Suplementar 2). Somente dois estudos definiram hipertensão pulmonar por diferentes pontos de cortes para PSAP: um acima de 40 mmHg,^12^ e um acima de 45 mmHg.^13^ O número de pacientes com disfunção ventricular esquerda ou direita foi estimado usando a FEVE média e desvio padrão respectivo (ou intervalo de confiança de 95%), conforme recomendado previamente para extração de dados nas revisões sistemáticas. Todos os dados foram organizados em uma tabela usando o programa Excel.

### Fontes hipotéticas de heterogeneidade

Devido à variação das características dos pacientes entre os estudos, esperamos uma heterogeneidade importante entre eles. Decidimos avaliar a gravidade da doença usando a proporção de indivíduos em ventilação mecânica e história de DCV (IC ou doença arterial coronariana). Utilizamos a estatística I ^2^ para identificar heterogeneidade, e meta-regressão, adotando essas características como potenciais modificadores dos achados ecocardiográficos anormais.

### Avaliação de qualidade

Usamos uma ferramenta descrita anteriormente para avaliar a qualidade metodológica de estudos observacionais, adaptada para relatos de caso e séries de casos.^14^ Para cada estudo, os revisores responderam as seguintes questões para avaliar se preenchiam os critérios de qualidade:

- Seleção: Os pacientes representam toda a experiência do investigador (centro) ou o método de seleção não é suficientemente claro, de modo que outros pacientes com apresentação similar podem não ter sido estudados?
- Determinação da exposição: A exposição foi definida adequadamente?
- Exclusão das causas alternativas: Outras causas alternativas que poderiam explicar a observação foram excluídas?
- Seguimento adequado: A ecocardiografia foi realizada no momento mais crítico do paciente durante sua internação?
- Detalhamento suficiente: O caso é descrito com detalhamento suficiente para que outros investigadores possam replicar a pesquisa ou outros profissionais possam fazer inferências em suas práticas?

Foram utilizados gráficos de funil para avaliar viés de publicação, uma vez que publicações de relatos de casos de achados cardíacos anormais são mais comuns. Os gráficos foram construídos traçando tamanho da amostra *versus* log odds ratio de cada desfecho, conforme proposto anteriormente na avaliação de viés de publicação de estudos não comparativos de proporções.^15^

### Análise estatística

Conduzimos uma metanálise de proporções para estimar a proporção de disfunção sistólica do VE, disfunção sistólica do VD, hipertensão pulmonar e efusão pericárdica em pacientes com Covid-19 aguda. Para avaliar se DCV prévia e a gravidade da doença influenciaram a proporção de achados ecocardiográficos anormais, realizamos uma meta-regressão usando a prevalência de DCV e a proporção de pacientes em ventilação mecânica em cada estudo. O nível de significância adotado foi p<0,05.

Dada a variabilidade esperada nos estudos selecionados, realizamos uma metanálise de efeitos aleatórios com transformação do arco seno duplo de Freeman-Tukey para detectar qualquer violação da presunção de normalidade nessa variável. A heterogeneidade foi avaliada pela estatística I^2^. A metanálise foi realizada usando o programa Stata ( *StataCorp* . College Station, Texas) versão 15.0.

## Resultados

### Resultados da busca

**Figura 1:**
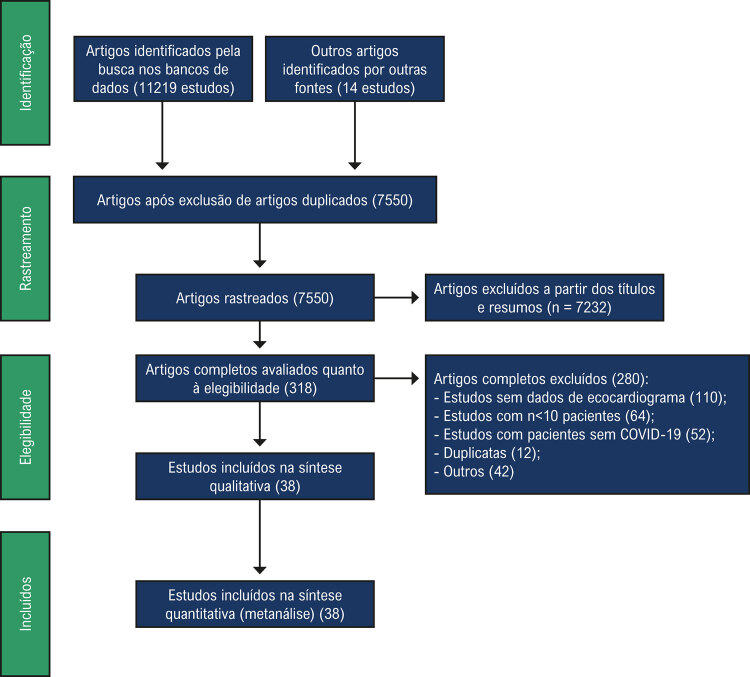
Fluxograma da seleção dos estudos para a metanálise.

A busca inicial resultou em 11 233 títulos, e o número final após exclusão dos artigos duplicados foi 7550 ( Figura 1 ). Desses, 318 eram estudos potencialmente relevantes e os respectivos textos completos foram analisados quanto à elegibilidade, e 38 estudos foram considerados elegíveis e incluídos na metanálise ( Tabela 1 ).

**Tabela 1:** Características dos estudos incluídos na metanálise

Primeiro autor (Mês Ano)	País	Características da população	Amostra com ecocardiograma	Idade média	Homens, %	Obesidade %	Hipertensão %	Diabetes, %	DCV prévia, %	IC prévia,%	ECO anormal prévio,% %	Ventilação mecânica%
Deng (Março2020) ^8^	China	Pacientes internados com Covid-19	112	65	51	37	32	17	13	4	4	25
Li (Abril2020) ^16^	China	Pacientes com Covid-19 com ecocardiograma	120	61	48	18	40	12	9	0	0	13
Bangalore (Abril2020) ^29^	EUA	Pacientes com Covid-19 com ecocardiograma	17	63	83	NR	61	33	17	NR	50	67
Rath (Maio2020) ^18^	Alemanha	Pacientes internados com Covid-19	98	68	63	20	70	24	23	NR	NR	40
Ge (Maio2020) ^30^	China	Pacientes com Covid-19 na UTI	51	70	73	NR	43	31	31	8	NR	41
Evrard (Maio2020) ^31^	França	Pacientes com Covid-19 em ventilação mecânica	18	70	67	NR	61	22	NR	NR	NR	100
Szekely (Maio2020) ^19^	Israel	Pacientes internados com Covid-19	100	66,1	63	29	57	29	16	7	2	10
Stefanini (Junho2020) ^32^	Itália	Pacientes com Covid-19 com STEMI	28	68	71	4	71	32	21	NR	NR	0
Dweck (Junho2020) ^9^	69 países	Pacientes com Covid-19 com ecocardiograma	1216	62	69	NR	37	19	20	9	NR	0
Vasudev (Junho2020) ^33^	EUA	Pacientes com Covid-19 com ecocardiograma	45	61,4	51	NR	64	56	27	24	9	NR
Lazzeri (Julho2020) ^13^	Itália	Pacientes internados com Covid-19	28	61	79	61	89	39	29	NR	NR	86
Rodríguez-Santamarta (Julho2020) ^34^	Espanha	Pacientes com Covid-19 na UTI	37	67,6	92	NR	NR	NR	5	0	NR	NR
van den Heuvel (Julho2020) ^17^	Holanda	Pacientes internados com Covid-19	51	63	80	0	41	18	22	0	18	33
Stöbe (Agosto2020) ^35^	Alemanha	Pacientes com Covid-19 com ecocardiograma	18	64	78	NR	72	28	11	NR	NR	78
Giustino (Agosto2020) ^36^	EUA	Pacientes internados com Covid-19	118	66	100	NR	NR	NR	NR	NR	NR	NR
Krishnamoorthy (Agosto2020) ^37^	EUA	Pacientes com Covid-19 com ecocardiograma	12	57	42	42	58	33	17	NR	NR	42
Schott (Agosto2020) ^38^	EUA	Pacientes com Covid-19 com ecocardiograma	66	60	58	86	58	35	NR	11	6	35
Sud (Agosto2020) ^22^	EUA	Pacientes com Covid-19 com ecocardigrama	24	64,5	54	NR	NR	NR	NR	8	8	42
Duerr (Setembro2020) ^39^	Alemanha	Pacientes internados com Covid-19	19	69	47	NR	63	26	37	NR	NR	NR
Kunal (Outubro2020) ^40^	Índia	Pacientes com Covid-19 sintomáticos	28	51	65	NR	38	32	13	1	NR	23
Lassen (Outubro2020) ^26^	Dinamarca	Pacientes com Covid-19 com ecocardiograma	214	69	55	18	57	24	16	10	NR	0
Jain (Outubro2020) ^20^	EUA	Pacientes com Covid-19 com ecocardiograma	77	61	7	5	6	4	2	2	NR	5
Lairez (Outubro2020) ^41^	França	Pacientes internados com Covid-19	31	57	87	23	48	32	NR	NR	NR	68
Weckbach (Novembro2020) ^42^	Alemanha	Pacientes internados com Covid-19 e lesão no miocárdio	18	70	89	NR	78	39	39	6	NR	50
Argulian (Novembro2020) ^43^	EUA	Pacientes internados com Covid-19	105	66	61	NR	NR	NR	NR	NR	NR	28
Gonzalez (Dezembro2020) ^44^	Portugal	Covid-19 Pacientes na UTI com Covid-19	30	61	NR	53	73	30	NR	NR	NR	23
Ferrante (Dezembro2020) ^45^	Itália	Pacientes com Covid-19 com TC de tórax	21	67	71	NR	54	21	15	NR	NR	20
Bagate (Dezembro2020) ^12^	França	Pacientes na UTI com Covid-19	67	61	82	31	54	36	NR	10	NR	99
Shmueli (Janeiro2021) ^46^	EUA	Pacientes com Covid-19 com ecocardiograma	60	66,2	65	17	47	27	17	13	NR	32
Moody (Janeiro2021) ^47^	Reino Unido	Pacientes com Covid-19 com ecocardiograma	164	61	78	NR	41	32	13	NR	NR	73
Pishgahi (Fevereiro2021) ^48^	Irã	Pacientes com Covid-19 com ecocardiograma	680	55	63	NR	44	25	16	NR	NR	NR
Morin (Março2021) ^49^	EUA	Pacientes com Covid-19 com ecocardiograma	396	67	48	NR	58	31	NR	NR	NR	21
Norderfeldt (Março2021) ^50^	Suécia	Pacientes com Covid-19 na UTI	67	58	94	NR	NR	NR	NR	NR	NR	100
Li (Março2021) ^51^	China	Pacientes internados com Covid-19	157	62	50	15	45	15	17	3	NR	24
Liaqat (Março2021) ^52^	Paquistão	Pacientes internados com Covid-19	181	44	59	5	17	17	NR	NR	NR	28
Mercedes (Abril2021) ^53^	República Dominicana	Gestantes com Covid-19	15	29	0	33	0	NR	0	0	NR	0
Karagodin (Maio2021) ^54^	10 países	Pacientes internados com Covid-19	870	60	56	NR	43	20	14	7	NR	27
Barberato (Julho2021) ^23^	Brasil	Pacientes internados com Covid-19	223	61,4	59	27	52	35	13	7	NR	NR

*Todos os estudos adotaram um nível de significância de 5% para teste de hipóteses. UTI: Unidade de Terapia Intensiva.*

### Achados ecocardiográficos nos pacientes com Covid-19

**Figura 2:**
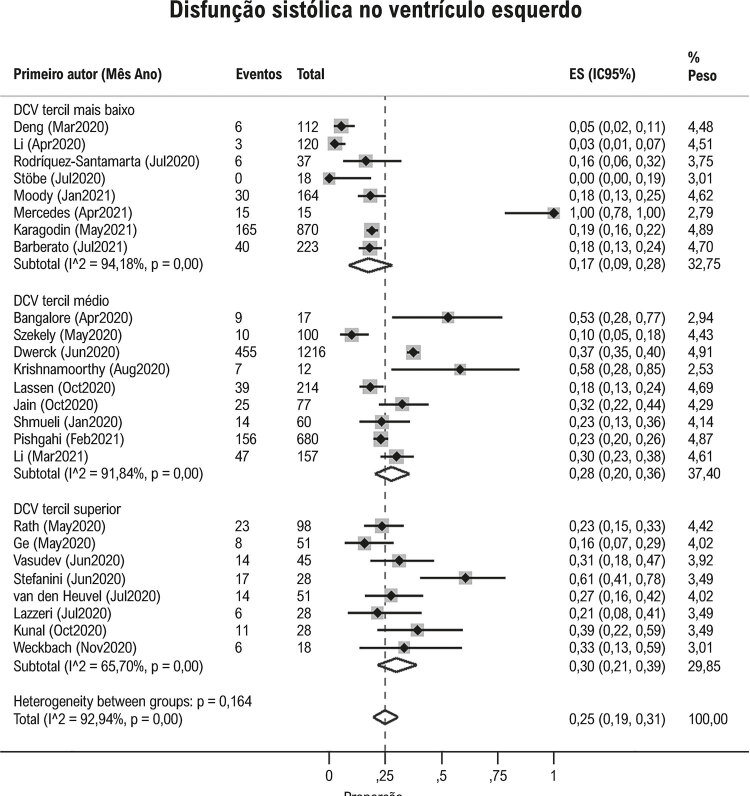
Proporção de disfunção ventricular esquerda em pacientes com Covid-19 nos estudos, de acordo com a prevalência de doenças cardiovasculares. DCV: doenças cardiovasculares. * Os estudos foram divididos de acordo com a porcentagem de pacientes com DCV: tercil mais alto (menos que 15%), tercil médio (15-21%) e tercil superior (>21%).

No geral, a proporção de disfunção sistólica do VE foi 25% (IC95%: 19, 31; I^2^93%; Figura 2 ), embora a heterogeneidade entre os estudos tenha sido alta. Tal heterogeneidade não pôde ser explicada nem pela prevalência de DCV prévia de cada estudo ( Figura 2 , p para interação = 0,16), nem pela proporção de cada estudo dos pacientes em ventilação mecânica (Figura Suplementar 1, p para interação = 0,37). Entre os estudos que apresentaram dados ecocardiográficos antes da infecção por SARS-CoV2, encontramos uma relação direta entre ecocardiograma anormal prévio e proporções de disfunção do VE (Figura Suplementar 3, p para interação < 0,001).

**Figura 3:**
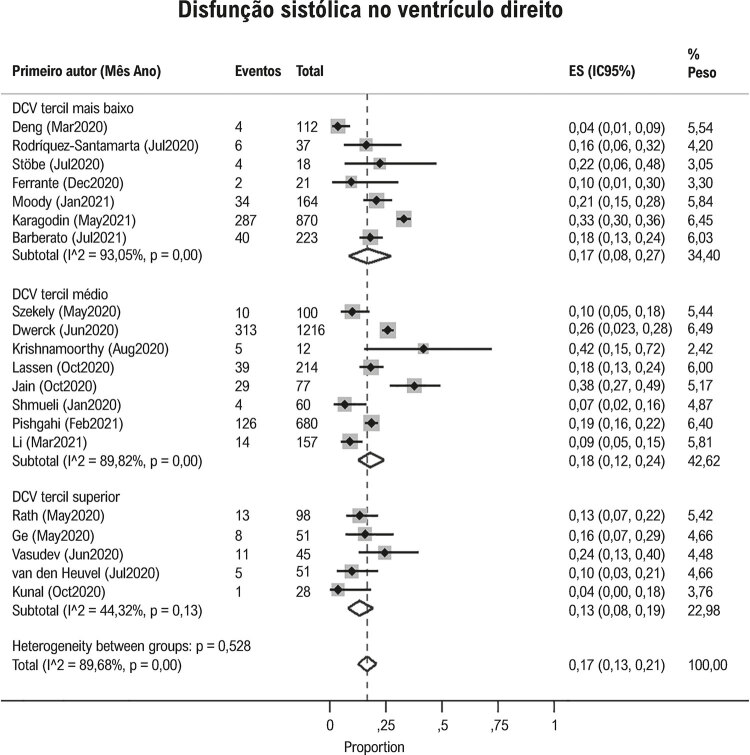
Proporção de disfunção ventricular direita em pacientes com Covid-19 nos estudos, de acordo com a prevalência de doenças cardiovasculares. DCV: doenças cardiovasculares. * Os estudos foram divididos de acordo com a porcentagem de pacientes com DCV: tercil mais alto (menos que 15%), tercil médio (15-21%) e tercil superior (>21%).

Disfunção sistólica do VD esteve presente em 17% (IC95% 13, 21; I^2^90%; Figura 3 ) dos pacientes com Covid-19. Contudo, apesar da alta heterogeneidade, DCV prévia (p=0,53), hipertensão pulmonar (p=0,96) e ventilação mecânica (p=0,65) não explicaram a variação na proporção de disfunção do VD entre os estudos ( Figura 3 , Figuras Suplementares 2 e 4).

**Figura 4:**
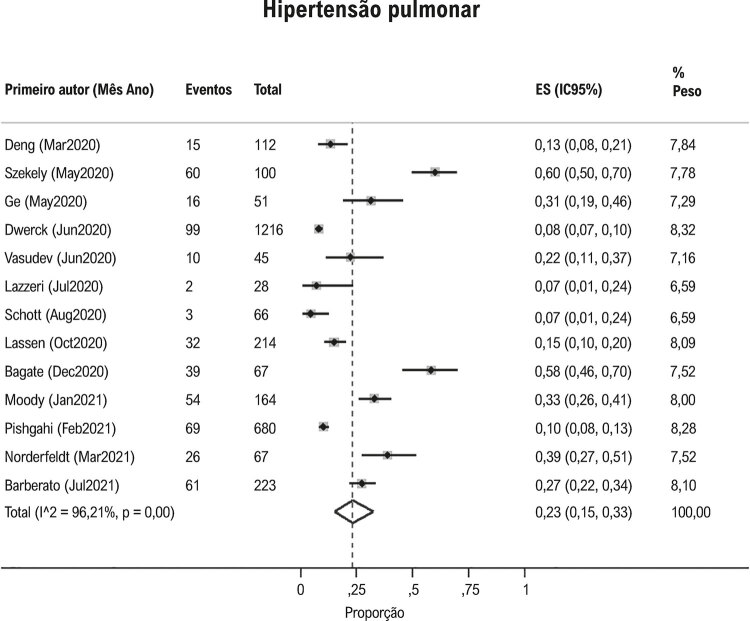
Proporção de hipertensão pulmonar em pacientes com Covid-19.

**Figura 5:**
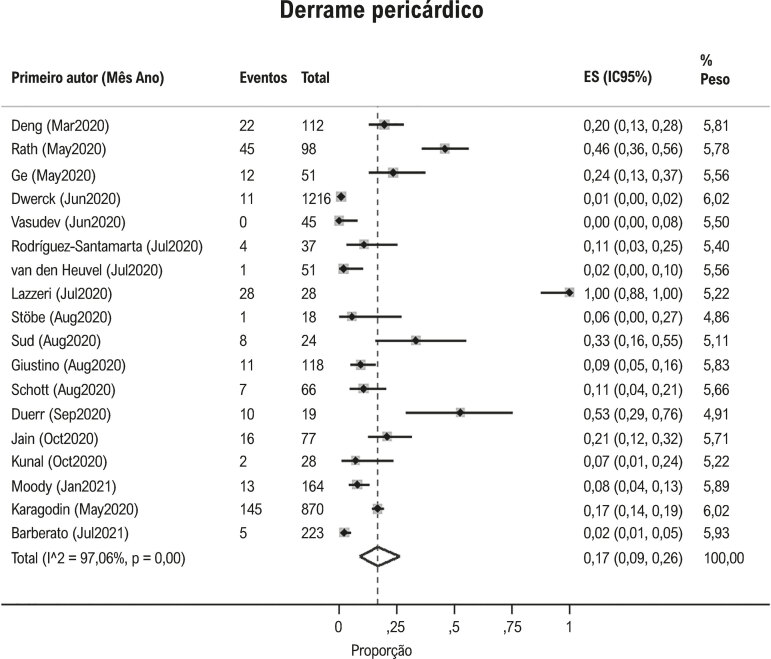
Proporção de derrame pericárdico em pacientes com Covid-19.

**Figura 6:**
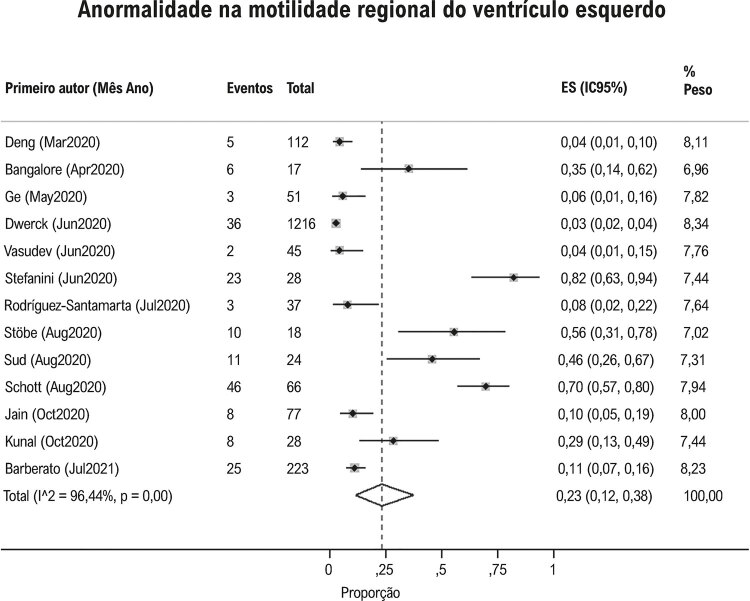
Proporção de anormalidade na motilidade regional do ventrículo esquerdo em pacientes com Covid-19.

**Figura 7:**
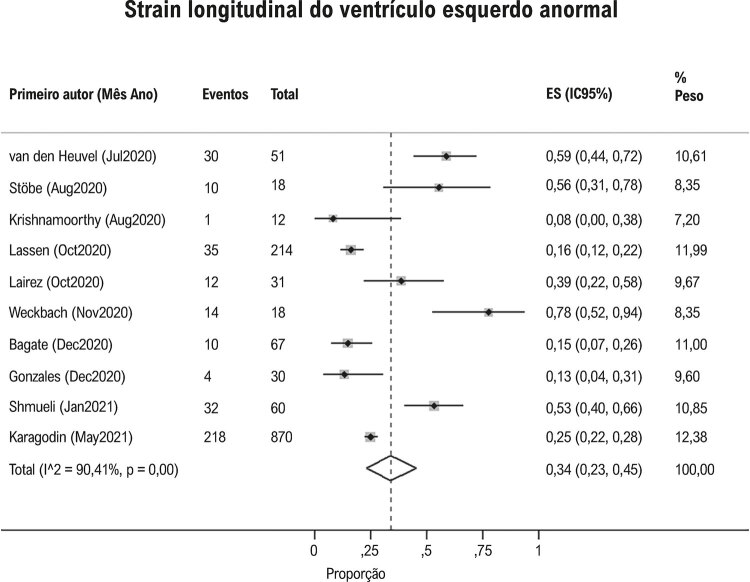
Proporção de strain longitudinal global do ventrículo esquerdo anormal em pacientes com Covid-19.

Hipertensão pulmonar foi observada em 23% (IC95%: 15, 33, I^2^ 96%; Figura 4 ) e derrame pericárdico em 17% (IC95%: 9, 26; I^2^97%; Figura 5 ) dos pacientes com Covid-19. Contratilidade segmentar anormal da parede do VE foi relatado em 23% (IC95% 12, 38; I^2^ 96%; Figura 6 ) em pacientes com Covid-19. O GLS foi anormal em 34% (IC95% 23, 45; I^2^90%) dos pacientes com Covid-19 *(*Figura 7 ).

### Viés de publicação

Avaliamos potencial viés de publicação de estudos relatando disfunção sistólica do VE, disfunção sistólica do VD, derrame pericárdico, e hipertensão pulmonar. A análise visual do gráfico de funil sugere viés de publicação dos estudos relatando disfunção sistólica do VD, com uma maior probabilidade de estudos pequenos relatarem uma maior proporção do desfecho (Figura Suplementar 5).

## Discussão

Nesta revisão sistemática de achados ecocardiográficos em pacientes com Covid-19, encontramos que as proporções estimadas de disfunção sistólica do VE foram 25%, disfunção sistólica do VD 17%, hipertensão pulmonar 23%, e derrame pericárdico 17%. GLS, que é mais sensível para detectar disfunção ventricular esquerda subclínica, foi anormal em 34% dos pacientes com Covid-19. Independente do método, os achados da disfunção sistólica variaram consideravelmente, com menores proporções em estudos relatando proporcionalmente menos indivíduos com ecocardiograma anormal prévio.

Os achados ecocardiográficos em pacientes com Covid-19 têm sido bem heterogêneos. A prevalência de disfunção sistólica do VE, disfunção do VD, e dilatação do VD variou de 5,4^8^ a 37,4%,^9^ 3,6^8^ a 33%,^16^ e 0^17^ a 46.9%,^18^ respectivamente. Enquanto a maioria dos estudos destacaram a disfunção e/ou dilatação do VD como a alteração ecocardiográfica mais frequente,^19^ outros relataram a disfunção sistólica do VE como a alteração mais prevalente.^8 , 9^ Os resultados contraditórios acerca da prevalência e das consequências das alterações ecocardiográficas em pacientes com Covid-19 podem ser explicados por vários fatores. Amostras relativamente pequenas, viés de referência, protocolos diferentes de ETE, definições imprecisas das anormalidades ecocardiográficas, e diferenças nas características das populações, tais como proporção de pacientes em ventilação mecânica, e/ou DCV prévia, podem ter levado às conclusões amplamente variadas a respeito das manifestações cardíacas da Covid-19. Na busca por fontes de heterogeneidade, alguns pontos interessantes devem ser mencionados em nosso estudo. Quando separamos os estudos pela proporção de pacientes em ventilação mecânica (como um indicador de gravidade da doença), as proporções de disfunção do VE e do VD não mudaram. Quando analisamos uma população composta por indivíduos mais sadios (o tercil mais baixo de prevalência de DCV prévia), a proporção de pacientes com disfunção do VE tendeu a ser menor, mas essa diferença não foi estatisticamente significativa. Por outro lado, é possível que elevadas proporções de achados ecocardiográficos anormais no início da pandemia reflitam disfunção do VE prévia, uma vez que encontramos uma proporção mais alta de disfunção do VE nos estudos descrevendo proporcionalmente mais indivíduos com ecocardiograma prévio anormal. Uma análise do Registro Brasileiro de Ecocardiografia mostrou que os pacientes com Covid-19 sem DCV prévia tinham menor probabilidade de apresentar disfunção sistólica do VE que pacientes sem DCV prévia (13 vs. 34%, p <0,001).^23^

O estudo de Dweck et al.^9^ foi o primeiro (e o maior) a mostrar que anormalidades ecocardiográficas eram muito comuns em pacientes internados com Covid-19. Utilizando um inquérito online que coletou dados de 1216 pacientes (26% com DCV pré-existente) de 69 países, os autores encontraram que mais da metade dos pacientes (55%) apresentaram um ETE anormal. Os indivíduos com achados ecocardiográficos anormais eram mais velhos, e tinham uma maior prevalência de DCV pré-existente, IC ou doença valvular cardíaca. Disfunção sistólica do VE, de qualquer grau, foi diagnosticado em 37,4% dos indivíduos, e disfunção biventricular em 14,3%. Por outro lado, somente 3% tinham evidência de um novo infarto do miocárdio, 3% de miocardite, e 2% de achados sugestivos de síndrome de Takotsubo. O estudo foi limitado por viés de seleção, o que pode ter levado à superestimação dos achados cardíacos.

A fim de minimizar os vieses de referência, Szekely et al.^19^ realizaram sistematicamente ETE em 100 pacientes consecutivos internado por Covid-19, 43% deles com DCV prévia. Os autores encontraram que a disfunção/dilatação do VD foi a anormalidade mais frequente (39%), enquanto uma minoria (10%) apresentou disfunção sistólica do VE.^19^ Ainda, pacientes com Covid-19 com injúria miocárdica ou uma pior condição clínica não apresentaram diferença significativa na função sistólica do VE, mas apresentaram pior função do VD em comparação a pacientes sem injúria miocárdica ou com melhor condição clínica. A maior prevalência de disfunção do VD e pequena proporção de disfunção do VE também foram encontradas em outros estudos menores.^20 , 21 , 24^ Apesar de a maioria dos estudos desta metanálise não ter claramente identificado a presença de alterações ecocardiográficas pré-existentes, é possível que, em uma pequena parcela dos pacientes, a disfunção sistólica do VE reflita disfunção ventricular esquerda ”nova”, relacionada à Covid-19, particularmente naqueles pacientes sem DCV prévia. Além disso, anormalidades ecocardiográficas podem indicar a presença de doença cardíaca estável pré-existente que tenha piorado por causa de uma infecção por SARS-CoV. Assim, parece que a prevalência de disfunção cardíaca seja mais baixa que a sugerida no início da pandemia. Dados acerca do uso de ecocardiografia em pacientes internados com Covid-19, obtidos de estudos com delineamento, tamanho amostral e escore de gravidade variáveis, mostraram que achados ecocardiográficos normais foram relatados em 50% dos indivíduos, com menor alteração da FEVE.^25^ De fato, um estudo recente apresentou que disfunção do VE persistente não é comum após Covid-19: em pacientes com níveis elevados de troponina, ressonância magnética cardíaca realizada dois meses após infecção revelou disfunção sistólica do VE em somente 11% dos pacientes, embora um terço deles apresentasse achados sugestivos de miocardite.^6^

Uma vez que os principais esforços da comunidade científica visam prevenir as consequências graves à saúde da pandemia da Covid-19, tem sido um desafio equilibrar o uso de ecocardiografia a fim de prover cuidado médico de alta qualidade sem aumento no risco de transmissão cruzada entre profissionais da saúde e pacientes. Por outro lado, é importante enfatizar que a presença de disfunção cardíaca associa-se independentemente com um pior prognóstico em pacientes com Covid-19 grave.^26 , 27^ Parâmetros ecocardiográficos que identificam dano no miocárdio de modo mais precoce e preciso que parâmetros convencionais, tais como GLS bidimensional do VE ou VD, têm sido menos utilizados no contexto da Covid-19 devido às recomendações para se utilizar protocolos direcionados, que reduzem a exposição de profissionais da saúde à infecção. Nossa metanálise mostrou que estudos que avaliaram a função sistólica do VE com GLS detectaram uma maior proporção de pacientes com disfunção do VE em comparação a outros estudos que usaram a FEVE. Uma metanálise publicada recentemente mostrou que um GLS do VE e do VD mais baixos se associaram independentemente com pior desfecho na Covid-19.^28^

Nosso estudo tem limitações que merecem atenção. A maioria dos estudos estão sujeitos a viés de referência porque os ecocardiogramas foram realizados a critério do médico assistente, que pode ter superestimado a ocorrência de achados ecocardiográficos anormais. A maioria dos estudos teve um delineamento retrospectivo, exceto por um estudo prospectivo no qual ETT foi realizada em pacientes consecutivos internados por Covid-19, independentemente da indicação clínica.^19^ Ainda, as características dos pacientes e a apresentação da Covid-19 variaram entre os estudos, resultando em uma heterogeneidade considerável. Embora tenhamos avaliado algumas fontes de heterogeneidade, essa manteve-se elevada em alguns subgrupos. Aspectos técnicos relacionados à ecocardiografia, que potencialmente levam a um viés de classificação, e diferentes definições de anormalidades cardíacas podem ser fontes de heterogeneidade. Por exemplo, a avaliação à beira do leito da função do VD e hipertensão pulmonar pode ser limitada em pacientes críticos. Além disso, a maioria dos estudos não relataram a presença de anormalidades cardíacas prévias nem se os achados ecocardiográficos eram novos ou não. Por fim, devido à restrição de idioma em nossa pesquisa, uma possível exclusão de artigos importantes que não foram publicados em português, inglês ou espanhol não pode ser afastada.

## Conclusão

Em pacientes internados com Covid-19, achados ecocardiográficos anormais indicando disfunção do VE foram relatados em um em cada quatro pacientes. Foram detectadas prevalências mais baixas de disfunção do VD e efusão pericárdica, embora a disfunção sistólica do VE possa estar relacionada à doença cardíaca prévia. De fato, encontramos uma associação direta entre ecocardiograma anormal prévio e as frequências de disfunção do VE no subgrupo de estudos que relataram resultados de ecocardiografia prévios, o que fornece insights que podem ajudar no planejamento de estudos ecocardiográficos na Covid-19.

## * Material suplementar

Para informação adicional, por favor, clique aqui.


