# Supplementary material for: Achados Ecocardiográficos Anormais em Pacientes Internados com Covid-19: Uma Revisão Sistemática e Metanálise
Source: Arq Bras Cardiol. 2022 Jul 7;119(2):267–79. [Article in Portuguese] doi: 10.36660/abc.20210485 (PMC9363071; doi:10.36660/abc.20210485)
Supplement: Supplementary file 1 [file 2021-0485-Supplemental-material.pdf]

Supplemental material

Supplemental Table 1: Quality assessment of studies included in the meta-analysis

First author (Month)	Selection	Exposure ascertainment	Outcome ascertainment	Alternative cause ruled out	Enought follow-up	Sufficient detail
Deng(Mar2020)	*	*	*	*	*	*
Li(Apr2020)	*	*	*	*	*	*
Bangalore(Apr2020)		*			*	
Rath(May2020)	*	*	*		*	*
Ge(May2020)	*	*				*
Evrard(May2020)	*	*	*		*	
Szekely(May2020)	*	*	*	*	*	*
Dweck(Jun2020)	*		*	*		*
Vasudev(Jun2020)		*	*	*		*
Stefanini(Jun2020)		*			*	*
Lazzeri(Jul2020)		*	*			
van den Heuvel(Jul2020)	*	*	*	*		*
Rodríguez-Santamarta(Jul2020)	*	*	*	*		
Giustino(Aug2020)		*				
Krishnamoorthy(Aug2020)	*	*	*			
Sud(Aug2020)		*	*			
Schott(Aug2020)	*	*	*	*		*
Stöbe(Aug2020)		*	*			*
Duerr(Sep2020)		*	*		*	
Kunal(Oct2020)	*	*				
Lairez(Oct2020)	*	*	*			*
Jain(Oct2020)	*	*	*	*	*	*
Lassen(Oct2020)	*	*	*	*	*	*
Weckbach(Nov2020)	*	*	*	*	*	
Argulian(Nov2020)			*			
Ferrante(Dec2020)	*	*	*			
Bagate(Dec2020)	*	*	*		*	*
Gonzales(Dec2020)	*	*	*	*	*	*
Moody(Jan2021)	*	*	*		*	*
Shmueli(Jan2021)	*	*	*			*
Pishgahi(Feb2021)		*	*		*	
Norderfeldt(Mar2021)		*	*		*	
Liaqat(Mar2021)		*	*		*	
Morin(Mar2021)		*	*		*	*
Li(Mar2021)		*	*			
Mercedes(Apr2021)		*	*		*	
Karagodin(May2021)	*	*	*	*		*

Barberato(Jul2021)	*	*			*	*

Stars indicates the study adequately fulfilled the following quality criteria, respectively:
Selection: Does the patient(s) represent(s) the whole experience of the investigator (center) or is the selection method unclear to the extent that other patients with similar presentation may not have been reported?
Exposure ascertainment: Was the exposure adequately ascertained?
Alternative cause ruled out: Were other alternative causes that may explain the observation ruled out?
Enough follow-up: The echocardiography was performed in the most critical moment during the patient hospitalization?
Sufficient detail: Is the case(s) described with sufficient details to allow other investigators to replicate the research or to allow practitioners make inferences to their own practice?

Supplemental Table 2. Study specific definitions of abnormal echocardiographic findings.

First author (Month Year)	LV systolic dysfunction	RV dysfunction	Pulmonary hypertenstion	Pericardial abnormality
Deng (Mar2020)	LVEF<50%	TAPSE < 16mm	peak TRV > 2.8 m/s and/or PAT < 105 ms	Pericardial effusion
Bangalore(Apr2020)	Definition NR	Data NR	Data NR	Data NR
Li (Apr2020)	LVEF < 53%	Data NR	Data NR	Data NR
Rath (May2020)	LVEF d50%	TAPSE < 20 mm, FAC < 35%	Data NR	Pericardial effusion
Szekely (May2020)	LVEF d50%	TAPSE < 17 mm, “s” wave < 9.5 cm/s, FAC < 35%	PAT < 100 ms	Data NR
Evrard(May2020)	Definition NR	Data NR	Data NR	Data NR
Ge(May2020)	LVEF<50%	TAPSE < 17mm	PSAP > 40 mmHg	Pericardial effusion
Dweck (Jun2020)	LVEF<55%	Definition NR	Definition NR	Pericardial effusion, cardiac tamponade
Vasudev (Jun2020)	LVEF <50%	Definition NR	Definition NR	Pericardial effusion
Stefanini (Jun2020)	LVEF <50%	Data NR	Data NR	Data NR
Rodríguez-Santamarta (Jul2020)	LVEF<50%	Definition NR	Data NR	Pericardial effusion
Lazzeri (Jul2020)	LVEF < 50%	Data NR	PASP > 45 mmHg	Pericardial effusion (all without hemodynamic significance)
van den Heuvel (Jul2020)	LVEF<52% or LV GLS < 18%	TAPSE < 17 mm, “s” wave < 9.5 cm/s	Data NR	Pericardial effusion
Krishnamoorthy(Aug2020)	LVEF <50%	Definition NR	Data NR	Data NR
Schott (Aug2020)	LVEF < 52	TAPSE < 17 mm, “s” wave < 9.5 cm/s	Definition NR	Small pericardial effusion
Sud(Aug2020)	Data NR	Definition NR	Data NR	Pericardial effusion
Giustino(Aug2020)	Data NR	TAPSE < 17 mm, “s” wave < 9.5 cm/s	Data NR	Pericardial effusion (mild, moderate, large)
Stöbe (Aug2020)	LVEF < 52%	TAPSE < 17 mm, RVLS < 23%	Data NR	Pericardial effusion
Duerr(Sep2020)	Data NR	Data NR	Data NR	Pericardial effusion
Lairez (Oct2020)	LVEF ≤ 50%	TAPSE < 17mm	Data NR	Data NR
Kunal(Oct2020)	Definition NR	RV systolic dysfunction	Data NR	Pericardial effusion
Jain (Oct2020)	LVEF ≤ 50%	TAPSE < 17 mm or visual assessment	Data NR	Trace Pericardial effusion

Lassen (Oct2020)	LVEF < 40%	TAPSE < 16mm	Definition NR	Data NR
Weckbach(Nov2020)	Definition NR	Data NR	Data NR	Data NR
Argulian(Nov2020)	Data NR	RV dilation and visual assessment	Data NR	Data NR
Bagate (Dec2020)	LVEF < 45%	TAPSE < 17 mm, “s” wave < 9.5 cm/s	PASP > 40 mmHg	Data NR
Ferrante(Dec2020)	LVEF < 50%	TAPSE<17 mm	PASP > 35 mmHg	Data NR
Gonzales(Dec2020)	LVEF <50%	TAPSE < 17 mm, “s” wave < 9.5 cm/s, FAC < 35%	Data NR	Data NR
Moody(Jan2021)	Visual assessment	TAPSE < 17 mm, FAC < 35%	peak TRV > 2.8 m/s	PERICARDIAL EFFUSION
Shmueli(Jan2021)	LVEF < 50%	TAPSE < 17 mm	peak TRV > 2,8 m/s	Data NR
Pishgahi (Feb2021)	LVEF < 50%	Data NR	PASP > 35 mmHg	Data NR
Norderfeldt (Mar2021)	LVEF < 50%	TAPSE < 17 mm, “s” wave < 9.5 cm/s, FAC < 35%	PASP > 35 mmHg	Data NR
Liaqat(Mar2021)	LVEF < 60	TAPSE < 17 mm	Data NR	Data NR
Li(Mar2021)	LVEF < 50%	TAPSE < 17 mm, “s” wave < 9.5 cm/s, FAC < 35%	Data NR	Data NR
Morin(Mar2021)	LVEF < 40%	Data NR	Data NR	Data NR
Mercedes(Apr2021)	LVEF < 50	Data NR	Data NR	Data NR
Karagodin(May2021)	LVEF < 50%, LVGLS > 19%	RV LS > 20%	Data NR	Data NR
Barberato(Jul2021)	LVEF < 50%	TAPSE < 17 mm, “s” wave < 9.5 cm/s	PASP > 35 mmHg	Pericardial effusion

First author (Month Year)	LV systolic dysfunction	RV dysfunction
Deng (Mar2020)	LVEF<50%	TAPSE < 16mm
Bangalore(Apr2020)	Definition NR	Data NR
Li (Apr2020)	LVEF < 53%	Data NR
Rath (May2020)	LVEF d50%	TAPSE < 20 mm, FAC < 35%
Szekely (May2020)	LVEF d50%	TAPSE < 17 mm, “s” wave < 9.5 cm/s, FAC < 35%
Evrard(May2020)	Definition NR	Data NR
Ge(May2020)	LVEF<50%	TAPSE < 17mm
Dweck (Jun2020)	LVEF<55%	Definition NR
Vasudev (Jun2020)	LVEF <50%	Definition NR
Stefanini (Jun2020)	LVEF <50%	Data NR
Rodríguez-Santamarta (Jul2020)	LVEF<50%	Definition NR

Lazzeri (Jul2020)	LVEF < 50%	Data NR
van den Heuvel (Jul2020)	LVEF<52% or LV GLS < 18%	TAPSE < 17 mm, “s” wave < 9.5 cm/s
Krishnamoorthy(Aug2020)	LVEF <50%	Definition NR
Schott (Aug2020)	LVEF < 52	TAPSE < 17 mm, “s” wave < 9.5 cm/s
Sud(Aug2020)	Data NR	Definition NR
Giustino(Aug2020)	Data NR	TAPSE < 17 mm, “s” wave < 9.5 cm/s
Stöbe (Aug2020)	LVEF < 52%	TAPSE < 17 mm, RVLS < 23%
Duerr(Sep2020)	Data NR	Data NR
Lairez (Oct2020)	LVEF ≤ 50%	TAPSE < 17mm
Kunal(Oct2020)	Definition NR	RV systolic dysfunction
Jain (Oct2020)	LVEF ≤ 50%	TAPSE < 17 mm or visual assessment
Lassen (Oct2020)	LVEF < 40%	TAPSE < 16mm
Weckbach(Nov2020)	Definition NR	Data NR
Argulian(Nov2020)	Data NR	RV dilation and visual assessment
Bagate (Dec2020)	LVEF < 45%	TAPSE < 17 mm, “s” wave < 9.5 cm/s
Ferrante(Dec2020)	LVEF < 50%	TAPSE<17 mm
Gonzales(Dec2020)	LVEF <50%	TAPSE < 17 mm, “s” wave < 9.5 cm/s, FAC < 35%
Moody(Jan2021)	Visual assessment	TAPSE < 17 mm, FAC < 35%
Shmueli(Jan2021)	LVEF < 50%	TAPSE < 17 mm
Pishgahi (Feb2021)	LVEF < 50%	Data NR
Norderfeldt (Mar2021)	LVEF < 50%	TAPSE < 17 mm, “s” wave < 9.5 cm/s, FAC < 35%
Liaqat(Mar2021)	LVEF < 60	TAPSE < 17 mm
Li(Mar2021)	LVEF < 50%	TAPSE < 17 mm, “s” wave < 9.5 cm/s, FAC < 35%
Morin(Mar2021)	LVEF < 40%	Data NR
Mercedes(Apr2021)	LVEF < 50	Data NR
Karagodin(May2021)	LVEF < 50%, LVGLS > 19%	RV LS > 20%
Barberato(Jul2021)	LVEF < 50%	TAPSE < 17 mm, “s” wave < 9.5 cm/s

LV – left ventricular, LVEF – LV ejection fraction, RV – right ventricular, TAPSE - tricuspid annular plane systolic excursion TRV – tricuspid regurgitation velocity, NR – not reported, PAT – pulmonar acceleration time, PASP – pulmonar artery systolic pressure
Data NR means that the data was not reported in that study; Definition NR means that data was reported without specifying the definition criteria for that data.

Supplemental Table 3. Search strategy according to database and respective number of retrieved titles

DATABASE	COVID-19	AND	CVD	Retrieved in 21/07/2021
**Pubmed**	“COVID-19” [Supplementary Concept] or 2019 novel coronavirus disease[tiab] or COVID19[tiab] or COVID-19 pandemic[tiab] or SARS-CoV-2 infection[tiab] or COVID-19 virus disease[tiab] or 2019 novel coronavirus infection[tiab] or 2019-nCoV infection[tiab] or coronavirus disease 2019[tiab] or coronavirus disease-19[tiab] or 2019-nCoV disease[tiab] or COVID-19 virus infection[tiab] or COVID-19[tiab] or SARS-CoV-2[tiab] or "Coronavirus Infections"[Mesh] or Coronavirus Infection[tiab] o Infection, Coronavirus[tiab] or Infections, Coronavirus[tiab] or Coronavirus[tiab]<br><br>Filters: Full text; Humans; English; Portuguese; Spanish		(("Cardiomegaly"[Mesh] OR "Myocardial Infarction"[Mesh] OR "Heart Arrest"[Mesh] OR "Heart Aneurysm"[Mesh] OR "Myocardial Ischemia"[Mesh] OR "Coronary Disease"[Mesh] OR "Heart Valve Diseases"[Mesh] OR "Heart Septal Defects"[Mesh] OR "Heart Rupture"[Mesh] OR "Heart Injuries"[Mesh] OR "Heart Failure"[Mesh] OR "Heart Diseases"[Mesh] OR "Heart"[Mesh] OR "Heart Failure, Diastolic"[Mesh] OR "Heart Failure, Systolic"[Mesh] OR "Heart Transplantation"[Mesh] OR "Cardiomyopathies"[Mesh] OR "Myocarditis"[Mesh] OR "Endocarditis"[Mesh] OR "Cardiomegaly"[Title/abstract] OR "Myocardial Infarction"[Title/abstract] OR "Heart Arrest"[Title/abstract] OR "Heart Aneurysm"[Title/abstract] OR "Myocardial Ischemia"[Title/abstract] OR "Coronary Disease"[Title/abstract] OR "Heart Valve Diseases"[Title/abstract] OR "Heart Septal Defects"[Title/abstract] OR "Heart Rupture"[Title/abstract] OR "Heart Injuries"[Title/abstract] OR "Heart Failure"[Title/abstract] OR "Heart Diseases"[Title/abstract] OR "Heart"[Title/abstract] OR "Heart Failure, Diastolic"[Title/abstract] OR "Heart Failure, Systolic"[Title/abstract] OR "Heart Transplantation"[Title/abstract] OR "Cardiomyopathies"[Title/abstract] OR "Myocarditis"[Title/abstract] OR "Endocarditis"[Title/abstract]) AND ((humans[Filter]) AND (english[Filter] OR portuguese[Filter] OR spanish[Filter]))) OR (Echocardiography [Mesh] OR "Transthoracic Echocardiography" OR "Echocardiography, Transthoracic" OR "Echocardiography, Cross-Sectional" OR "Echocardiography, Cross Sectional" OR "Cross-Sectional Echocardiography" OR "Cross Sectional Echocardiography" OR "Echocardiography, M-Mode" OR "Echocardiography, M Mode" OR "M-Mode Echocardiography" OR "M Mode Echocardiography" OR "Echocardiography, Contrast" OR "Contrast Echocardiography" OR "2D Echocardiography" OR "Echocardiography, Two-Dimensional" OR "Echocardiography, Two Dimensional" OR "Echocardiography, 2-D" OR "Echocardiography, 2 D" OR "Two-Dimensional Echocardiography" OR "Two Dimensional Echocardiography" OR "2-D Echocardiography" OR "2	9

			D Echocardiography" OR "Echocardiogram" AND ((humans[Filter]) AND (english[Filter] OR portuguese[Filter] OR spanish[Filter])))	
**Lilacs and Scielo preprints**	**tw:(covid-19 OR "2019 novel coronavirus disease" OR "COVID-19 pandemic" OR "SARS-CoV-2 infection" OR "COVID-19 virus disease" OR "2019 novel coronavirus infection" OR "2019-nCoV infection" OR "coronavirus disease 2019" OR "coronavirus disease-19" OR "2019-nCoV disease" OR "COVID-19 virus infection" OR "SARS-CoV-2" OR "Coronavirus Infection" OR "Infection, Coronavirus" OR "Infections, Coronavirus" OR coronavirus)**<br><br>**Filters: LILACS, Scielo preprints, English, Spanish, Portuguese (#AND (db:("PREPRINT-SCIELO" OR "LILACS") AND la:("en" OR "pt"))**<br><br>#		tw:("Cardiomegaly" OR "Myocardial Infarction" OR "Heart Arrest" OR "Heart Aneurysm" OR "Myocardial Ischemia" OR "Coronary Disease" OR "Heart Valve Diseases" OR "Heart Septal Defects" OR "Heart Rupture" OR "Heart Injuries" OR "Heart Failure" OR "Heart Diseases" OR "Heart" OR "Heart Failure, Diastolic" OR "Heart Failure, Systolic" OR "Heart Transplantation" OR "Cardiomyopathies" OR "Myocarditis" OR "Endocarditis") OR ("Transthoracic Echocardiography" OR "Echocardiography, Transthoracic" OR "Echocardiography, Cross-Sectional" OR "Echocardiography, Cross Sectional" OR "Cross-Sectional Echocardiography" OR "Cross Sectional Echocardiography" OR "Echocardiography, M-Mode" OR "Echocardiography, M Mode" OR "M-Mode Echocardiography" OR "M Mode Echocardiography" OR "Echocardiography, Contrast" OR "Contrast Echocardiography" OR "2D Echocardiography" OR "Echocardiography, Two-Dimensional" OR "Echocardiography, Two Dimensional" OR "Echocardiography, 2-D" OR "Echocardiography, 2 D" OR "Two-Dimensional Echocardiography" OR "Two Dimensional Echocardiography" OR "2-D Echocardiography" OR "2 D Echocardiography" OR "Echocardiogram")	46

Embase	**('coronavirus disease 2019'/exp OR 'coronavirus disease 2019':ti,ab OR '2019-ncov disease':ti,ab OR '2019-ncov infection':ti,ab OR 'covid 19':ti,ab OR 'covid 2019':ti,ab OR 'ncov 2019 disease':ti,ab OR 'ncov 2019 infection':ti,ab OR 'novel coronavirus 2019 disease':ti,ab OR 'novel coronavirus 2019 infection':ti,ab OR 'novel coronavirus disease 2019':ti,ab OR 'novel coronavirus infection 2019':ti,ab OR 'wuhan coronavirus disease':ti,ab OR 'wuhan coronavirus infection':ti,ab) AND ([english]/lim OR [portuguese]/lim OR [spanish]/lim) AND [humans]/lim AND [embase]/lim**		('cardiomegaly'/exp OR 'myocardial infarction'/exp OR 'heart arrest'/exp OR 'heart aneurysm'/exp OR 'myocardial ischemia'/exp OR 'coronary disease'/exp OR 'heart valve diseases'/exp OR 'heart septal defects'/exp OR 'heart rupture'/exp OR 'heart injuries'/exp OR 'heart failure'/exp OR 'heart diseases'/exp OR 'heart'/exp OR 'heart failure, diastolic'/exp OR 'heart failure, systolic'/exp OR 'heart transplantation'/exp OR 'cardiomyopathies'/exp OR 'myocarditis'/exp OR 'endocarditis'/exp OR 'cardiomegaly':ti,ab OR 'myocardial infarction':ti,ab OR 'heart arrest':ti,ab OR 'heart aneurysm':ti,ab OR 'myocardial ischemia':ti,ab OR 'coronary disease':ti,ab OR 'heart valve diseases':ti,ab OR 'heart septal defects':ti,ab OR 'heart rupture':ti,ab OR 'heart injuries':ti,ab OR 'heart failure':ti,ab OR 'heart diseases':ti,ab OR 'heart':ti,ab OR 'heart failure, diastolic':ti,ab OR 'heart failure, systolic':ti,ab OR 'heart transplantation':ti,ab OR 'cardiomyopathies':ti,ab OR 'myocarditis':ti,ab OR 'endocarditis':ti,ab) AND ([embase]/lim OR [medline]/lim) AND ([english]/lim OR [portuguese]/lim OR [spanish]/lim) AND [humans]/lim AND [embase]/lim<br><br>OR<br><br>('echocardiography'/exp OR 'transthoracic echocardiography' OR 'echocardiography, transthoracic' OR 'echocardiography, cross-sectional' OR 'echocardiography, cross sectional' OR 'cross-sectional echocardiography' OR 'cross sectional echocardiography' OR 'echocardiography, m-mode' OR 'echocardiography, m mode' OR 'm-mode echocardiography' OR 'm mode echocardiography' OR 'echocardiography, contrast' OR 'contrast echocardiography' OR '2d echocardiography' OR 'echocardiography, two-dimensional' OR 'echocardiography, two dimensional' OR 'echocardiography, 2-d' OR 'echocardiography, 2 d' OR 'two-dimensional echocardiography' OR 'two dimensional echocardiography' OR '2-d echocardiography' OR '2 d	<

			echocardiography' OR 'echocardiogram') AND ([english]/lim OR [portuguese]/lim OR [spanish]/lim) AND [humans]/lim AND [embase]/lim	
**Cochrane (CENTRAL)**	#1 MeSH descriptor: [Coronavirus] explode all trees<br>#2 MeSH descriptor: [Coronavirus Infections] explode all trees<br>#3 (COVID-19 or "2019 novel coronavirus disease" or "COVID-19 pandemic" or "SARS-CoV-2 infection" or "COVID-19 virus disease" or "2019 novel coronavirus infection" or "2019-nCoV infection" or "coronavirus disease 2019" or "coronavirus disease-19" or "2019-nCoV disease" or "COVID-19 virus infection" or "SARS-CoV-2" or "Coronavirus Infection" or "Infection, Coronavirus" or "Infections, Coronavirus" or Coronavirus):ti,ab,kw<br>#4 #1 OR #2 OR #3		#5 MeSH descriptor: [Cardiomegaly] explode all trees<br>#6 MeSH descriptor: [Myocardial Infarction] explode all trees<br>#7 MeSH descriptor: [Heart Arrest] explode all trees<br>#8 MeSH descriptor: [Heart Aneurysm] explode all trees<br>#9 MeSH descriptor: [Myocardial Ischemia] explode all trees<br>#10 MeSH descriptor: [Coronary Disease] explode all trees<br>#11 MeSH descriptor: [Heart Valve Diseases] explode all trees<br>#12 MeSH descriptor: [Heart Septal Defects] explode all trees<br>#13 MeSH descriptor: [Heart Rupture] explode all trees<br>#14 MeSH descriptor: [Heart Injuries] explode all trees<br>#15 MeSH descriptor: [Heart Failure] explode all trees<br>#16 MeSH descriptor: [Heart Diseases] explode all trees<br>#17 MeSH descriptor: [Heart Failure, Diastolic] explode all trees<br>#18 MeSH descriptor: [Heart Failure, Systolic] explode all trees<br>#19 MeSH descriptor: [Heart Transplantation] explode all trees<br>#20 MeSH descriptor: [Cardiomyopathies] explode all trees<br>#21 MeSH descriptor: [Myocarditis] explode all trees<br>#22 MeSH descriptor: [Endocarditis] explode all trees<br>#23 ("Cardiomegaly" OR "Myocardial Infarction" OR "Heart Arrest" OR "Heart Aneurysm" OR "Myocardial Ischemia" OR "Coronary Disease" OR "Heart Valve Diseases" OR "Heart Septal Defects" OR "Heart Rupture" OR "Heart Injuries" OR "Heart Failure" OR "Heart Diseases" OR "Heart" OR "Heart Failure, Diastolic" OR "Heart Failure, Systolic" OR "Heart Transplantation" OR "Cardiomyopathies" OR "Myocarditis" OR "Endocarditis"):ti,ab,kw<br>#24 {OR #5 -#23}<br>#25 MeSH descriptor: [Echocardiography] explode all trees<br>#26 ("Transthoracic Echocardiography" OR "Echocardiography, Transthoracic" OR "Echocardiography, Cross-Sectional" OR "Echocardiography, Cross Sectional" OR "Cross-Sectional Echocardiography" OR "Cross Sectional Echocardiography" OR "Echocardiography, M-Mode" OR "Echocardiography, M Mode" OR "M-Mode Echocardiography" OR	6

			"M Mode Echocardiography" OR "Echocardiography, Contrast" OR "Contrast Echocardiography" OR "2D Echocardiography" OR "Echocardiography, Two-Dimensional" OR "Echocardiography, Two Dimensional" OR "Echocardiography, 2-D" OR "Echocardiography, 2 D" OR "Two-Dimensional Echocardiography" OR "Two Dimensional Echocardiography" OR "2-D Echocardiography" OR "2 D Echocardiography" OR "Echocardiogram"):ti,ab,kw<br>#27 #25 OR #26<br>#28 #24 OR #27<br>#29 #28 AND #4	
**MedRxiv**	ABSTRACT:"(2019-nCoV" OR ABSTRACT:"2019nCoV" OR ABSTRACT:"COVID-19" OR ABSTRACT:"SARS-CoV-2" OR ABSTRACT:"COVID19" OR ABSTRACT:"COVID" OR ABSTRACT:"SARS-nCoV" OR ABSTRACT:"wuhan coronavirus" OR ABSTRACT:"Coronavirus" OR ABSTRACT:"Corona virus" OR ABSTRACT:"corona-virus" OR ABSTRACT:"corona viruses" OR ABSTRACT:"coronaviruses" OR ABSTRACT:"SARS-CoV" OR ABSTRACT:"coronavirus disease 2019" OR ABSTRACT:"novel		Cardiomegaly<br>Myocardial Infarction<br>Heart Arrest<br>Heart Aneurysm<br>Myocardial Ischemia<br>Coronary Disease<br>Heart Valve Diseases<br>Heart Septal Defects<br>Heart Rupture<br>echocardiography<br>transthoracic echocardiography<br>echocardiography cross-sectional<br>echocardiography, cross sectional<br>cross-sectional echocardiography<br>cross sectional echocardiography<br>echocardiography, m-mode<br>echocardiography, m mode<br>m-mode echocardiography<br>m mode echocardiography<br>echocardiography, contrast<br>contrast echocardiography<br>2d echocardiography<br>echocardiography, two-dimensional<br>echocardiography, two dimensional<br>echocardiography, 2-d<br>echocardiography, 2 d<br>two-dimensional echocardiography<br>two dimensional echocardiography	;

	coronavirus disease" OR ABSTRACT:"COVID-19 pandemic"		2-d echocardiography<br>2 d echocardiography<br>echocardiogram	
**TOTAL**				/ <

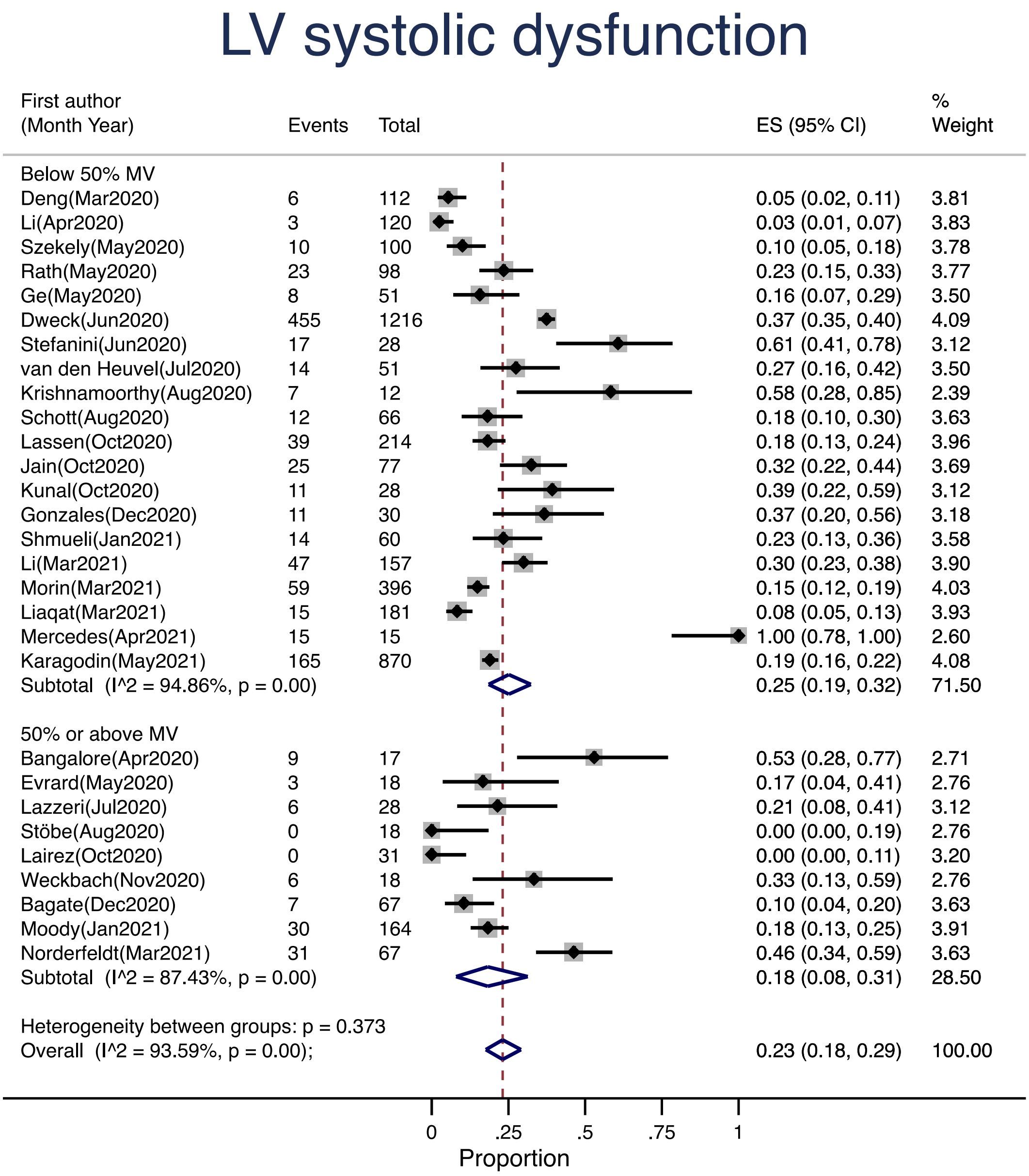

Figure 1 (suppl): Proportion of left ventricular dysfunction in patients with COVID-19 according to the proportion of individuals – below or above 50% - n mechanical ventilation (MV)

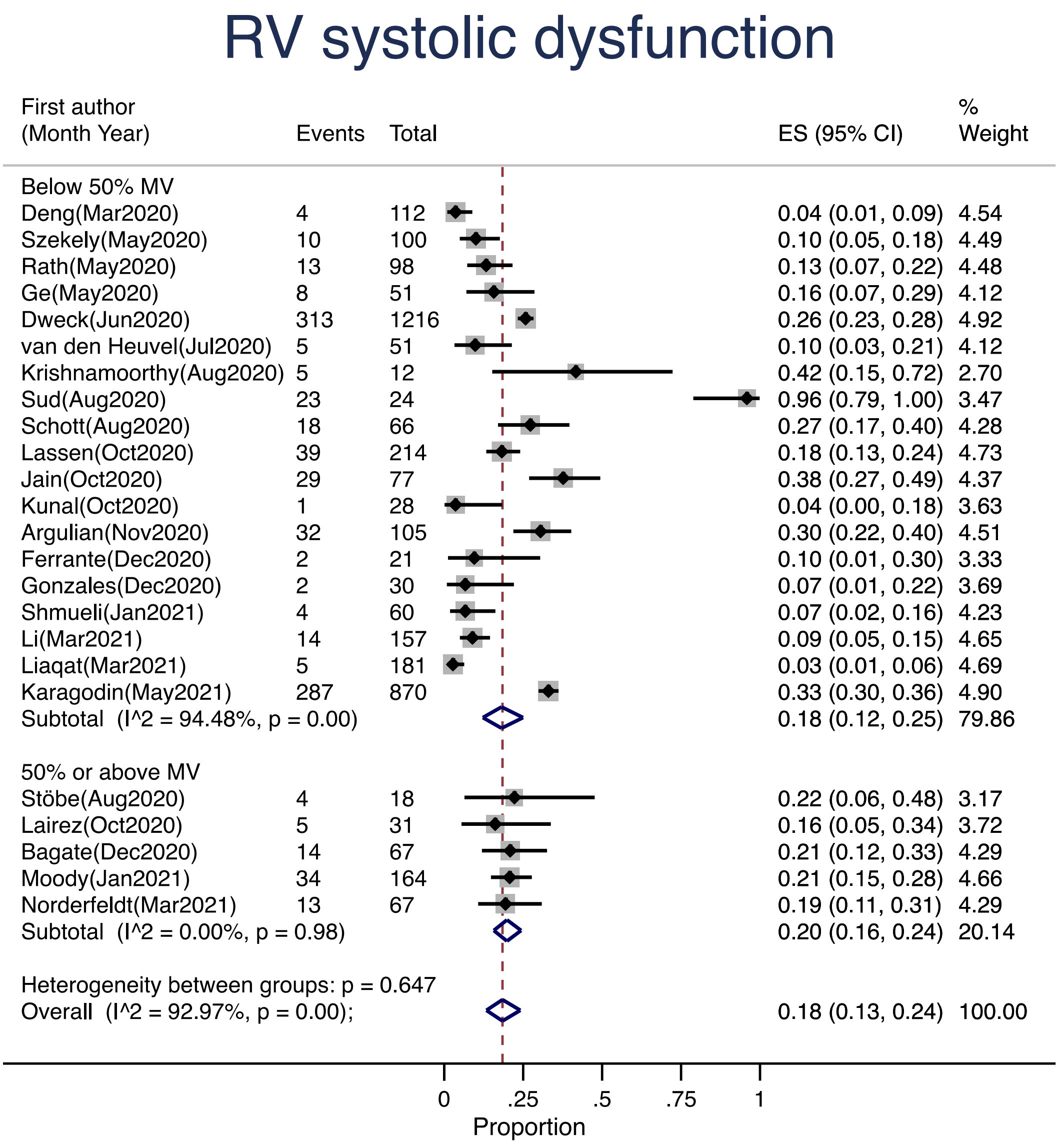

Figure 2 (suppl): Proportion of right ventricular dysfunction in patients with COVID-19 according to the proportion of individuals in mechanical ventilation (MV)

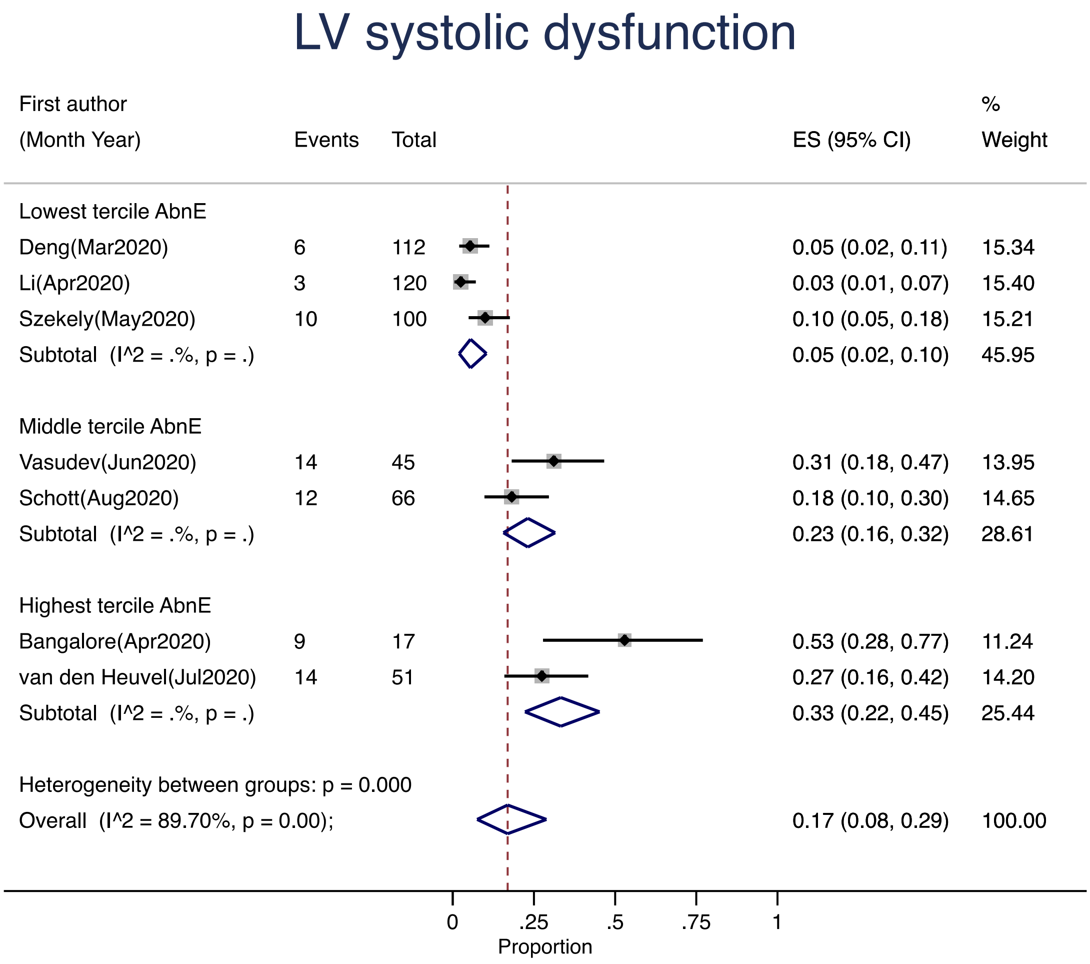

Figure 3 (suppl): Proportion of left ventricular dysfunction in patients with COVID-19 according to the proportion of previous abnormal echocardiogram (AbnE)
* Studies were divided according to study specific percentage of AbnE: Lowest tercile (less than 4%), Middle tercile (4 to 9%) and Highest tercile (9 to 53%)

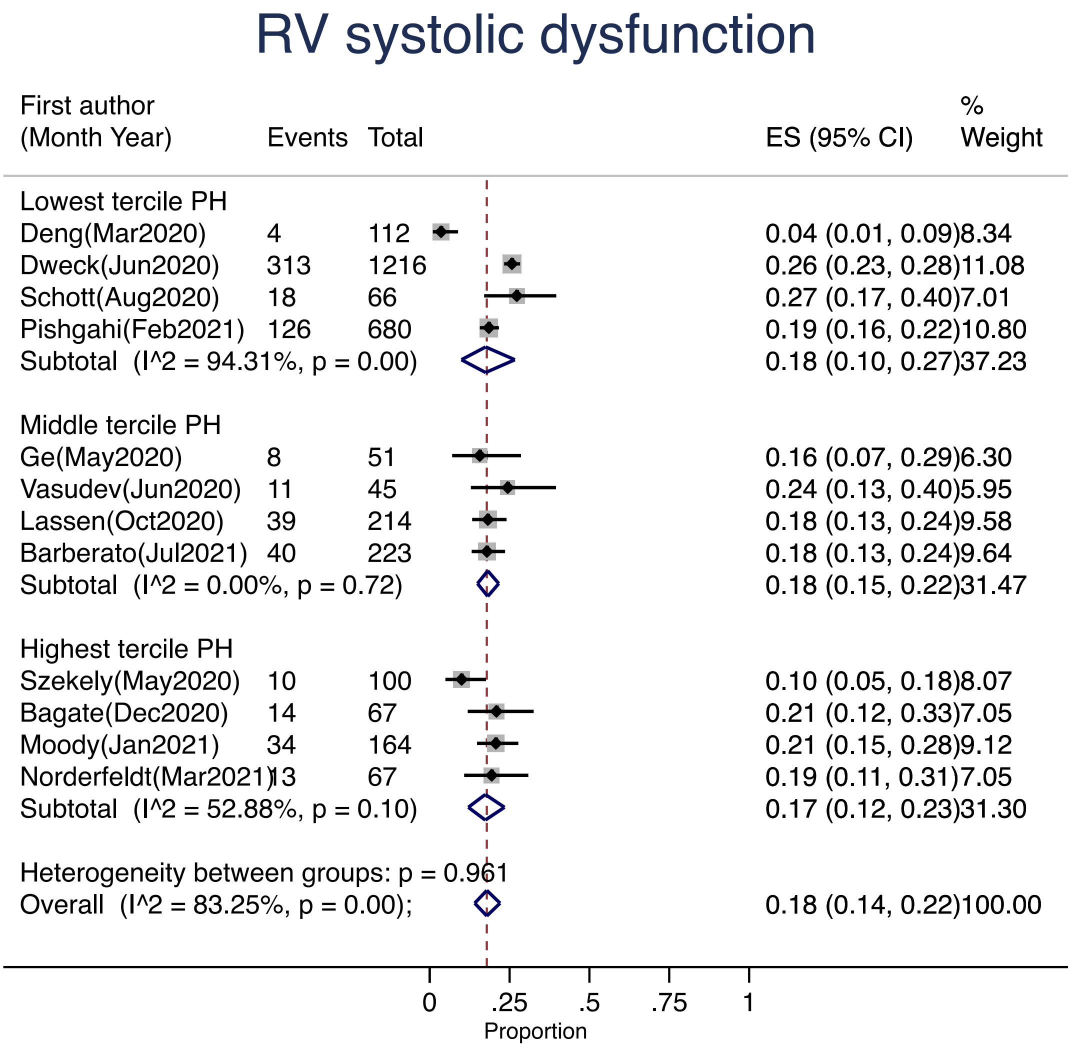

Figure 4 (suppl): Proportion of right ventricular dysfunction in patients with COVID-19 according to concomitant findings of pulmonary hypertension (PH)
* Studies were divided according to study specific percentage of PH: Lowest

tercile (less than 14%), Middle tercile (14 to 32%) and Highest tercile (>32%)

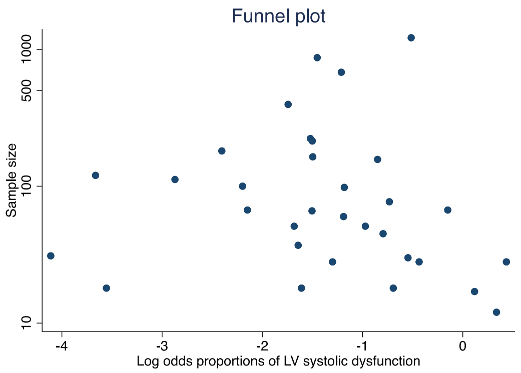
Funnel plot
Sample size
10
100
500
1000
-4
-3
-2
-1
0
Log odds proportions of LV systolic dysfunction

A.

B.

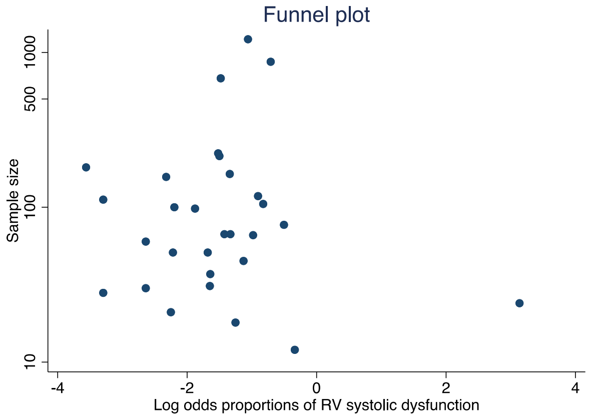
Funnel plot
Sample size
10
100
500
1000
-4
-2
0
2
4
Log odds proportions of RV systolic dysfunction

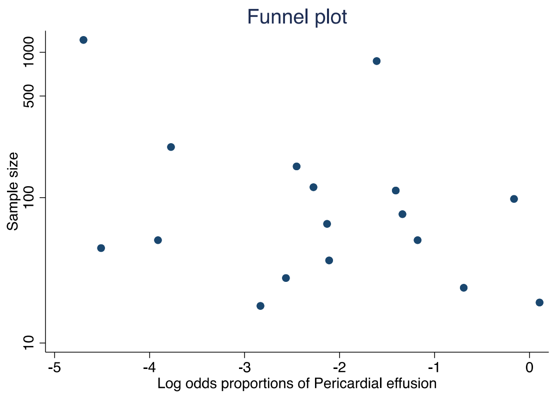
Funnel plot
Sample size
10
100
500
1000
-5
-4
-3
-2
-1
0
Log odds proportions of Pericardial effusion

C.

D.

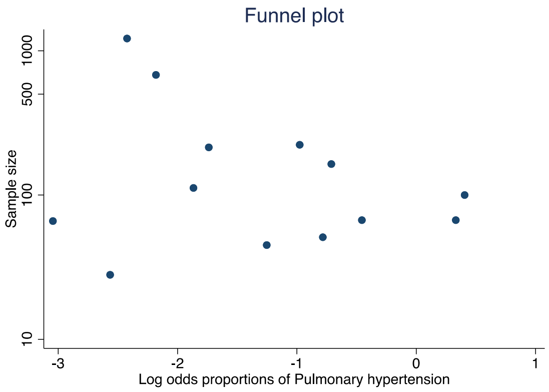
Funnel plot
Sample size
10
100
500
1000
-3
-2
-1
0
1
Log odds proportions of Pulmonary hypertension

Figure 5 (suppl): Funnel plot using study sample size and the log odds proportions of each main outcome: (A) LV systolic dysfunction, (B) RV systolic dysfunction, (C) pericardial effusion and (D) pulmonary hypertension[12].
Y axis is in log scale.
