# Supplementary material for: Achados Ecocardiográficos Anormais em Pacientes Internados com Covid-19: Uma Revisão Sistemática e Metanálise
Source: Arq Bras Cardiol. 2022 Jul 7;119(2):267–79. [Article in Portuguese] doi: 10.36660/abc.20210485 (PMC9363071; doi:10.36660/abc.20210485)
Supplement: Translation

# Translation: Abnormal Echocardiographic Findings in Hospitalized Patients with Covid-19: A Systematic Review and Meta-analysis

**Authors:** Silvio Henrique Barberato, Eduardo G. Bruneto, Gabriel S. Reis, Paula Rauen Franco de Oliveira, Alexandre F. Possamai, Odilson Silvestre, Miguel M. Fernandes Silva

**Affiliations:** 1 Brazil CardioEco Centro de Diagnóstico Cardiovascular, Curitiba, PR – Brazil; 2 Brazil Quanta Diagnóstico, Ecocardiografia,Curitiba, PR – Brazil; 3 Brazil Universidade Federal do Acre, Rio Branco, AC – Brazil; 4 Brazil Universidade Federal do Paraná, Curitiba, PR – Brazil; 5 Brazil Pontifícia Universidade Católica do Paraná, Curitiba, PR – Brazil

**Keywords:** Echocardiography, Covid-19, Ventricular Function, Left

## Abstract

**Background:**

Coronavirus disease 2019 (Covid-19) can lead to severe respiratory distress and acute cardiac injury, but it is unclear how often it can cause cardiac dysfunction.

**Objective:**

In this systematic review, we aimed to summarize the main echocardiographic findings in patients with Covid-19.

**Methods:**

We systematically searched in PUBMED, EMBASE, LILACS and Cochrane databases, in addition MedRxiv and Scielo preprints from inception to July 21^st^, 2021. Studies reporting echocardiographic data in patients with Covid-19 were included. Demographic characteristics, previous cardiovascular disease (CVD), and echocardiographic findings were extracted. We performed a meta-analysis of proportions to estimate the main echocardiographic findings. The level of significance was p < 0.05.

**Results:**

From 11,233 studies, 38 fulfilled inclusion criteria and were included in the meta-analysis. The estimated proportions of left ventricular (LV) systolic dysfunction were 25% (95%CI: 19, 31; I^2^93%), abnormal global longitudinal strain 34% (95% CI 23, 45; I^2^90%), righ ventricular (RV) systolic dysfunction 17% (95%CI 13, 21; I^2^90%), pericardial effusion 17% (95%CI: 9, 26; I^2^97%), and pulmonary hypertension 23% (95%CI: 15, 33, I2 96%). LV systolic dysfunction was directly associated with study-specific prevalence of previous abnormal echocardiogram (p<0.001). The proportion of patients in mechanical ventilation, indicating severity of disease, did not explain the heterogeneity in the proportions of LV dysfunction (p=0.37).

**Conclusion:**

Among hospitalized patients with Covid-19, LV dysfunction has been reported in one quarter, with smaller proportions of right ventricular dysfunction, pericardial effusion and pulmonary hypertension. However, there was a higher proportion of LV dysfunction among studies reporting the presence of prior heart disease, which suggests that cardiac dysfunction was mostly pre-existing.

## Introduction

Coronavirus disease 2019 (Covid-19) caused by the severe acute respiratory syndrome coronavirus 2 (SARS-CoV-2) may result in severe respiratory distress and acute cardiac injury. Elevated troponin blood levels and imaging showing abnormal cardiac function have been associated with worse prognosis in patients with acute Covid-19.^1^ The worse prognosis may result from a combination of disease-related factors, such as virulence and inflammatory response, and patient-related factors, such as pre-existent cardiovascular risk factors and established cardiovascular disease (CVD). For this reason, it has been recommended to assess cardiac function using transthoracic echocardiography (TTE) to guide the management of patients with new or worsening cardiovascular symptoms, hemodynamic instability, and increased biomarkers levels.^2^

At the beginning of the pandemic, there have been anecdotal reports of new-onset heart failure (HF) and fulminant myocarditis in patients with Covid-19.^3 , 4^ Studies using cardiovascular magnetic resonance have shown that evidence of myocardial inflammation in elite athletes who had recently recovered from Covid-19 was common, but with uncertain clinical significance.^5^ Nevertheless, more recent reports have shown that myocarditis is far less common (less than 2%) than earlier suggested, even among patients with elevated circulating troponin levels.^6 , 7^ It is also unclear how often Covid-19 affects cardiac function, either due to direct myocardial injury or through increasing pulmonary resistance when the lungs are severely damaged. Echocardiographic studies have shown varied findings of left and right ventricular dysfunction; for instance, left ventricular (LV) systolic dysfunction has been found in less than 10% of patients in some studies and almost 40% in others.^8 , 9^ Large-scale studies showing accurate estimates of the incidence of major cardiac dysfunction, its clinical significance, and associated risk factors are lacking. Because of the risk of contamination of healthcare workers, the use of echocardiography should be based on critical consideration of the benefits for the patient.^10^ Therefore, we aimed to summarize the main echocardiographic findings of patients with Covid-19 through a systematic review and meta-analysis.

## Methods

### Study design and eligibility criteria

We performed a systematic review, study selection and meta-analysis of proportions according with the PRISMA statement for meta-analysis.^11^ We included all studies with at least 10 participants describing echocardiogram findings in hospitalized patients with Covid-19, published in English, Portuguese, and Spanish languages, from inception to July 21, 2021. Studies that did not report any echocardiogram findings were excluded. We also excluded unpublished abstracts, studies lacking baseline clinical information of participants or insufficient echocardiographic data to obtain the number of participants with abnormal cardiac function or structure.

### Information sources and search

We systematically searched in PUBMED, EMBASE, LILACS and Cochrane (CENTRAL) databases. We also searched articles in the repository of unpublished (preprints) manuscripts in MedRxiv (https://www.medrxiv.org/) and Scielo preprint databases. Our search used the terms “Covid-19”, “SARS CoV 2”, “Coronavirus infection”, “Heart Diseases” and “Echocardiography” as descriptors (Medical Subject Headings – MeSH) or supplementary concept, and synonyms as free text in title and abstract to increase sensitivity. The full search strategy was displayed in the supplemental material (Supplemental table 3).

### Study selection and data extraction

We merged the search results from each database using the EndNote software and removed duplicated studies. Four authors (EB, GR, PO, AP) independently examined titles and abstracts to remove irrelevant reports. Then, the full texts of potentially relevant reports were examined and the studies that fulfilled the eligibility criteria were selected. Different reports from the same study were linked and the study with the largest sample size was selected. Discrepancies were resolved by consensus. References of review articles were examined for additional studies, and those considered eligible were further incorporated into the meta-analysis.

The following data were extracted from the studies: authors’ names, month of publication, previous abnormal echocardiogram of patients, sample size of patients undergoing echocardiogram, and the number of individuals with LV systolic dysfunction, right ventricular (RV) systolic dysfunction, pulmonary hypertension, and pericardial effusion. The study-specific definitions for each echocardiographic abnormality were detailed in the supplemental table 2. When LV systolic dysfunction was not clearly defined by the authors, we adopted a LV ejection fraction (LVEF) below 50%. Abnormal global longitudinal strain (GLS) was defined as below 18%. Similarly, RV dysfunction was determined by study specific definition; otherwise, it was defined as tricuspid annular plane systolic excursion (TAPSE) below 17 mm and/or tissue Doppler of the free lateral wall of the right ventricle (S’) below 9.5 cm/s. Pulmonary hypertension was defined by tricuspid regurgitation velocity above 2.8 m/s, pulmonary acceleration time below 100 ms and/or pulmonary artery systolic pressure (PASP) above 35 mmHg (Supplemental Table 2). Only two studies defined pulmonary hypertension by different cut off values of PASP: one above 40mmHg,^12^ and one above 45mmHg.^13^ The number of patients with LV or RV dysfunction was estimated using the mean LVEF and the respective standard deviation (or 95% confidence interval) as previously recommended for data extraction in systematic reviews.^14^

Population characteristics, including mean age, proportion of men, prevalence of obesity, hypertension, diabetes, previous coronary heart disease and heart failure were also extracted. All data were entered in a table using Excel software.

### Hypothesized sources of heterogeneity

Since patient characteristics varied among the studies, we expected a significant heterogeneity across them. We decided to evaluate severity of disease using the proportion of individuals under mechanical ventilation and history of previous CVD (either HF or coronary heart disease). We used I^2^statistics to identify heterogeneity and meta-regression using these characteristics as potential modifiers of abnormal echocardiographic findings.

### Quality assessment

We used a previously reported tool for evaluating methodological quality of observational studies, adapted for case reports and case series.^14^ For each study, the reviewers answered the following questions to evaluate whether they fulfilled the quality criteria:

- Selection: Does the patient(s) represent(s) the whole experience of the investigator (center) or is the selection method unclear to the extent that other patients with similar presentation may not have been reported?
- Exposure ascertainment: Was the exposure adequately ascertained?
- Alternative cause ruled out: Were other alternative causes that may explain the observation ruled out?
- Enough follow-up: The echocardiography was performed in the most critical moment during the patient hospitalization?
- Sufficient detail: Is the case described with sufficient details to allow other investigators to replicate the research or to allow practitioners make inferences to their own practice?

Funnel plots were used to assess for publication bias, as case reports of abnormal cardiac findings might be more likely to be published. Funnel plots were constructing by plotting sample size against log odds ratio of each outcome, as previously proposed to assess publication bias in meta-analysis of non-comparative proportion studies.^15^

### Statistical analysis

We performed a meta-analysis of proportions to estimate the proportion of LV systolic dysfunction, RV systolic dysfunction, pulmonary hypertension and pericardial effusion among patients with acute Covid-19. To assess whether previous CVD and severity of disease influenced the proportion of abnormal echo findings, we performed a meta-regression using the prevalence of CVD and the proportion of participants under mechanical ventilation in each study. The level of significance was p < 0.05.

Due to expected variability in the selected studies, we performed a random-effects meta-analysis with Freeman-Tukey double arc-sine transformation to account for any violation of the assumption of normality in this variable. Heterogeneity was assessed with the I^2^statistic. The meta-analysis was performed using Stata ( *StataCorp* . College Station, Texas) version 15.0.

## Results

### Search Results

The initial search yielded 11,233 titles, and the final number after exclusion of duplicates was 7,550 ( Figure 1 ). From these, 318 were potentially relevant studies and the respective full texts were assessed for eligibility. Finally, 38 studies met the eligibility criteria and were included in the meta-analysis ( Table 1*).*

**Figure 1:** Flow-chart of study selection for the meta-analysis.

**Table 1:** Characteristics of studies included in the meta-analysis

First author (Month Year)	Country	Population characteristics	Sample with echo	Mean age	Men, %	Obesity, %	Hypertension, %	Diabetes, %	Previous CVD, %	Previous HF, %	Previous Abnormal Echo, %	Mechanical ventilation, %
Deng (Mar2020) ^8^	China	hospitalized patients with Covid-19	112	65	51	37	32	17	13	4	4	25
Li (Apr2020) ^16^	China	Covid-19 patients with echocardiogram	120	61	48	18	40	12	9	0	0	13
Bangalore (Apr2020) ^29^	USA	Covid-19 patients with electrocardiogram	17	63	83	NR	61	33	17	NR	50	67
Rath (May2020) ^18^	Germany	hospitalized patients with Covid-19	98	68	63	20	70	24	23	NR	NR	40
Ge (May2020) ^30^	China	Covid-19 patients in the ICU	51	70	73	NR	43	31	31	8	NR	41
Evrard (May2020) ^31^	France	Covid-19 patients in mechanical ventilation	18	70	67	NR	61	22	NR	NR	NR	100
Szekely (May2020) ^19^	Israel	hospitalized patients with Covid-19	100	66.1	63	29	57	29	16	7	2	10
Stefanini (Jun2020) ^32^	Italy	Covid-19 patients with STEMI	28	68	71	4	71	32	21	NR	NR	0
Dweck (Jun2020) ^9^	69 countries	Presumed Covid-19 patients with echocardiogram	1216	62	69	NR	37	19	20	9	NR	0
Vasudev (Jun2020) ^33^	USA	Covid-19 patients with echocardiogram	45	61.4	51	NR	64	56	27	24	9	NR
Lazzeri (Jul2020) ^13^	Italy	hospitalized patients with Covid-19	28	61	79	61	89	39	29	NR	NR	86
Rodríguez-Santamarta (Jul2020) ^34^	Spain	Covid-19 patients in the ICU	37	67.6	92	NR	NR	NR	5	0	NR	NR
van den Heuvel (Jul2020) ^17^	Netherlands	hospitalized patients with Covid-19	51	63	80	0	41	18	22	0	18	33
Stöbe (Aug2020) ^35^	Germany	Covid-19 patients with echocardiogram	18	64	78	NR	72	28	11	NR	NR	78
Giustino (Aug2020) ^36^	USA	hospitalized patients with Covid-19	118	66	100	NR	NR	NR	NR	NR	NR	NR
Krishnamoorthy (Aug2020) ^37^	EUA	Covid-19 patients with echocardiogram	12	57	42	42	58	33	17	NR	NR	42
Schott (Aug2020) ^38^	USA	Covid-19 patients with echocardiogram	66	60	58	86	58	35	NR	11	6	35
Sud (Aug2020) ^22^	USA	Covid-19 patients with echocardiogram	24	64.5	54	NR	NR	NR	NR	8	8	42
Duerr (Sep2020) ^39^	Germany	hospitalized patients with Covid-19	19	69	47	NR	63	26	37	NR	NR	NR
Kunal (Oct2020) ^40^	India	symptomatic Covid-19 patients	28	51	65	NR	38	32	13	1	NR	23
Lassen (Oct2020) ^26^	Denmark	Covid-19 patients with echocardiogram	214	69	55	18	57	24	16	10	NR	0
Jain (Oct2020) ^20^	USA	Covid-19 patients with echocardiogram	77	61	7	5	6	4	2	2	NR	5
Lairez (Oct2020) ^41^	France	hospitalized patients with Covid-19	31	57	87	23	48	32	NR	NR	NR	68
Weckbach (Nov2020) ^42^	Germany	Covid-19 and myocardial injury	18	70	89	NR	78	39	39	6	NR	50
Argulian (Nov2020) ^43^	USA	hospitalized patients with Covid-19	105	66	61	NR	NR	NR	NR	NR	NR	28
Gonzales (Dec2020) ^44^	Portugal	Covid-19 patients in the ICU	30	61	NR	53	73	30	NR	NR	NR	23
Ferrante (Dec2020) ^45^	Italy	Covid-19 patients with chest-CT	21	67	71	NR	54	21	15	NR	NR	20
Bagate (Dec2020) ^12^	France	Covid-19 patients in the ICU	67	61	82	31	54	36	NR	10	NR	99
Shmueli (Jan2021) ^46^	USA	Covid-19 patients with echocardiogram	60	66.2	65	17	47	27	17	13	NR	32
Moody (Jan2021) ^47^	United Kingdom	Covid-19 patients with echocardiogram	164	61	78	NR	41	32	13	NR	NR	73
Pishgahi (Feb2021) ^48^	Iran	Covid-19 patients with echocardiogram	680	55	63	NR	44	25	16	NR	NR	NR
Morin (Mar2021) ^49^	USA	Covid-19 patients with echocardiogram	396	67	48	NR	58	31	NR	NR	NR	21
Norderfeldt (Mar2021) ^50^	Sweden	Covid-19 patients in the ICU	67	58	94	NR	NR	NR	NR	NR	NR	100
Li (Mar2021) ^51^	China	hospitalized patients with Covid-19	157	62	50	15	45	15	17	3	NR	24
Liaqat (Mar2021) ^52^	Pakistan	hospitalized patients with Covid-19	181	44	59	5	17	17	NR	NR	NR	28
Mercedes (Apr2021) ^53^	Dominican Republic	Covid-19 pregnant patients	15	29	0	33	0	NR	0	0	NR	0
Karagodin (May2021) ^54^	10 countries	hospitalized patients with Covid-19	870	60	56	NR	43	20	14	7	NR	27
Barberato (Jul2021) ^23^	Brazil	hospitalized patients with Covid-19	223	61.4	59	27	52	35	13	7	NR	NR

All studies adopted a 5% level of statistical significance for hypothesis testing. ICU: intensive care unit; NR: not reported.

### Echocardiographic findings in Covid-19 patients

Overall, we found that the proportion of LV systolic dysfunction was 25% (95%CI: 19, 31; I^2^93%; Figure 2 ), but heterogeneity was high across the studies. This heterogeneity was neither explained by study-specific prevalence of previous CVD ( Figure 2 , p for interaction = 0.16), nor by the study-specific proportion of patients under mechanical ventilation (Supplemental figure 1, p for interaction = 0.37). Among the studies that reported echocardiographic data before SARS-CoV2 infection, we found a direct relationship between previous abnormal echocardiogram and proportions of LV dysfunction (Supplemental Figure 3, p for interaction < 0.001).

**Figure 2:** Proportion of left ventricular dysfunction in patients with Covid-19 across the studies according to the prevalence of cardiovascular diseases. CVD: cardiovascular diseases. LV: left ventricular. * Studies were divided according to the percentage of patients with CVD: Lowest tercile (less than 15%), Middle tercile (15 to 21%) and the highest tercile (>21%).

RV systolic dysfunction was present in 17% (95%CI 13, 21; I^2^90%; Figure 3 ) of patients with Covid-19. However, despite the high heterogeneity, previous CVD (p=0.53), pulmonary hypertension (p=0.96), or mechanical ventilation (p=0.65) do not explain the variation in proportion of RV dysfunction across the studies ( Figure 3 , Supplemental Figures 2 and 4).

**Figure 3:** Proportion of right ventricular dysfunction in patients with Covid-19 across the studies according to the prevalence of cardiovascular diseases. CVD: cardiovascular diseases; RV: right ventricular. * Studies were divided according to the percentage of patients with CVD: Lowest tercile (less than 15), Middle tercile (15 to 21%) and Highest tercile (>21%).

Pulmonary hypertension was found in 23% (95%CI: 15, 33, I2 96%; Figure 4 ) and pericardial effusion was found in 17% (95%CI: 9, 26; I^2^97%; Figure 5 ) of patients with Covid-19. Abnormal regional LV wall motion were reported in 23% (95% CI 12, 38; I^2^96%; Figure 6 ) in Covid-19 patients. GLS was abnormal in 34% (95% CI 23, 45; I^2^90%) of patients with Covid-19 *(*Figure 7 ).

**Figure 4:** Proportion of pulmonary hypertension in patients with Covid-19.

**Figure 5:** Proportion of pericardial effusion in patients with Covid-19.

**Figure 6:** Proportion of regional LV motion abnormality in patients with Covid-19. LV: left ventricular.

**Figure 7:** Proportion of Abnormal LV global longitudinal strain in patients with Covid-19. LV: left ventricular; GLS: global longitudinal strain.

### Publication bias

We evaluated potential publication bias of studies reporting LV systolic dysfunction, RV systolic dysfunction, pericardial effusion, and pulmonary hypertension. Visual analysis of the funnel plot suggests publication bias of studies reporting RV systolic dysfunction, with a higher likelihood of small studies reporting a higher proportion of the outcome *(* Supplemental Figure 5 *).*

## Discussion

In this systematic review of echocardiographic findings in patients with Covid-19, we found that the estimated proportions of LV systolic dysfunction was 25%, RV systolic dysfunction was 17%, pulmonary hypertension was 23% and pericardial effusion was 17%. GLS, which is more sensitive to detect subclinical LV dysfunction, was abnormal in 34% of patients with Covid-19. Despite the method, the findings of LV systolic dysfunction varied considerably, with lower proportions in studies reporting proportionally fewer individuals with previous abnormal echocardiogram.

The echocardiographic findings in patients with Covid-19 have been very heterogeneous. The prevalence of LV systolic dysfunction, RV dysfunction and RV dilation have ranged from 5.4^8^ to 37.4%,^9^ 3.6^8^ to 33%,^16^ and 0^17^ to 46.9%,^18^ respectively. While most studies have pointed out RV dysfunction and/or dilation as the most frequent echocardiographic changes,^19 - 22^ others have found LV systolic dysfunction to be more prevalent.^8 , 9^ The contradictory results about the prevalence and consequences of echocardiographic changes among patients with Covid-19 may be explained by several factors. Relatively small samples, referral bias, different TTE protocols, inaccurate definitions of echocardiographic abnormalities, and differences of population characteristics, such as the proportion of patients on mechanical ventilation and/or with previous CVD, might have led to the wide-ranging conclusions about cardiac manifestations of Covid-19. In the search for sources of heterogeneity, some interesting points should be mentioned in our study. When we separated the studies by the proportion of patients under mechanical ventilation (as an indicator of disease severity), the proportions of LV and RV dysfunction did not change. When we analyzed a population composed of healthier individuals (the lowest tertile of prior CVD prevalence), the proportion of patients with LV dysfunction tended to be lower, but this difference was not statistically significant. On the other hand, it is conceivable that high proportions of abnormal echocardiographic findings at the beginning of the pandemic reflect previous LV dysfunction, as we found higher proportion of LV dysfunction in studies reporting proportionally more individuals with previous abnormal echocardiogram. An analysis from the Brazilian Echocardiographic Registry showed that patients with Covid-19 without previous CVD were less likely to have LV systolic dysfunction than those with previous CVD (13 vs 34%, p <0.001).^23^

The study by Dweck et al.^9^ was the first (and the largest) to show that echocardiographic abnormalities were very common in hospitalized Covid-19 patients. Using an online survey which collected data from 1,216 patients (26% with pre-existing CVD) of 69 countries, they found that more than half of the patients (55%) had an abnormal TTE. Subjects with abnormal echocardiographic findings were older, and had a higher prevalence of pre-existing CVD, HF or valvular heart disease. Any degree of LV systolic dysfunction was diagnosed in 37.4% of subjects and biventricular impairment in 14.3%. On the other hand, only 3% had evidence of a new myocardial infarction, 3% of myocarditis and 2% of findings suggestive of Takotsubo syndrome. The study was limited by selection bias, which might have led to the overestimation of cardiac findings.

In order to mitigate referral bias, Szekely et al.^19^ systematically performed TTE in 100 consecutive patients hospitalized for Covid-19, 43% of which had prior CVD. They found that the most frequent abnormality was RV dysfunction/dilation (39%) while only a minority of patients (10%) presented LV systolic dysfunction.^19^ In addition, Covid-19 patients with myocardial injury or worse clinical condition did not have any significant difference in LV systolic function but had worse RV function when compared to patients without myocardial injury or better clinical condition. The higher prevalence of RV dysfunction and small proportion of LV dysfunction have been similarly found in other smaller studies.^20 , 21 , 24^ Although most studies of this meta-analysis have not clearly identified the presence of pre-existing echocardiographic changes, it is possible that in a small proportion of patients, LV systolic dysfunction reflects a Covid-19-related “de novo” LV impairment, particularly in those without previous CVD. Moreover, echocardiographic abnormalities might denote the presence of pre-existing stable cardiac disease that has worsened because of the SARS-CoV-2 infection. Therefore, it appears that the prevalence of cardiac dysfunction is lower than that suggested at the beginning of the pandemic. Data regarding the use of echocardiography on hospitalized Covid-19 patients, retrieved from studies with variable designs, sample sizes, and severity scores, have shown that normal echocardiographic findings were reported in about 50% of subjects, with LVEF usually less affected.^25^ Indeed, it has been recently shown that persistent LV dysfunction is uncommon after Covid-19: in patients who had elevated troponin blood levels, cardiovascular magnetic resonance two months after infection revealed LV systolic dysfunction in only 11% of patients, although one-third had findings suggestive of myocarditis.^6^

Since the major efforts of the scientific community aim to prevent the severe health consequences of the Covid-19 pandemic, it has been challenging to balance the use of echocardiography to provide high-quality medical care without an increase in the risk of cross-infection between healthcare professionals and patients. On the other hand, it is important to emphasize that the presence of cardiac dysfunction is independently associated with worse prognosis in patients with severe Covid-19.^26 , 27^ Echocardiographic parameters that identify myocardial damage earlier and more accurately than the traditional ones, such as two-dimensional LV or RV GLS, have been less used in the context of Covid-19 due to the recommendations for using focused protocols, which reduces the exposure of health care professionals to infection. Our meta-analysis showed that studies that assessed LV systolic function with GLS detected a higher proportion of patients with LV dysfunction compared to those that used LVEF. A recently published meta-analysis showed that lower LV and RV GLS were independently associated with poor outcome in Covid-19.^28^

Our study has limitations that deserve attention. Most studies are subject to referral bias because echocardiograms were performed at the discretion of the attending physician, which may have overestimated the occurrence of abnormal echocardiographic findings. Most studies had a retrospective design, except for one prospective study in which TTE was performed in consecutive patients hospitalized for Covid-19, regardless of clinical indication.^19^ Moreover, population characteristics and presentation of Covid-19 varied across studies, resulting in considerable heterogeneity. Although we explored a few sources of heterogeneity, heterogeneity remained high within subgroups. Echocardiogram-related technical aspects, leading to potential misclassification bias, and different definitions of cardiac abnormalities may be additional sources of heterogeneity. For instance, bedside evaluation of RV function and pulmonary hypertension may be limited in critical ill patients. Also, most studies did not report the presence of prior cardiac abnormalities nor whether the echocardiographic findings were new. Finally, because of language restriction in our search, possible exclusion of relevant papers that were not published in Portuguese, English or Spanish may not be excluded.

## Conclusion

In hospitalized patients with Covid-19, abnormal echocardiographic findings indicating LV dysfunction have been reported in one of four patients. Lower prevalence of RV dysfunction and pericardial effusion was detected, although LV systolic dysfunction may be related to prior heart disease. Indeed, we found a direct association between previous abnormal echocardiogram and the proportions of LV dysfunction in the subgroup of studies that reported previous echocardiogram, which provide insights that help plan echocardiographic studies in Covid-19.

## * Material suplementar

For additional information, please click here
